# State-controlled epidemic in a game against a novel pathogen

**DOI:** 10.1038/s41598-022-19691-7

**Published:** 2022-09-20

**Authors:** József Garay, Ádám Kun, Zoltán Varga, Manuel Gámez, Ana Belén Castaño-Fernández, Tamás F. Móri

**Affiliations:** 1 Institute of Evolution, Centre for Ecological Research, Konkoly-Thege M. út 29-33, Budapest, 1121 Hungary; 2 MTA-ELTE Theoretical Biology and Evolutionary Ecology Research Group and Department of Plant Systematics, Ecology and Theoretical Biology, Eötvös Loránd University, Pázmány Péter sétány 1/c, 1117 Budapest, Hungary; 3 Department of Mathematics and Modelling, Institute of Mathematics and Basic Science, Hungarian University of Agriculture and Life Sciences, Páter K. u. 1., 2100 Gödöllő, Hungary; 4 Department of Mathematics, University of Almería, La Cañada de San Urbano, 04120 Almería, Spain; 5 Alfréd Rényi Institute of Mathematics, Reáltanoda u. 13-15., Budapest, 1053 Hungary

**Keywords:** Evolutionary theory, Applied mathematics

## Abstract

The pandemic reminded us that the pathogen evolution still has a serious effect on human societies. States, however, can prepare themselves for the emergence of a novel pathogen with unknown characteristics by analysing potential scenarios. Game theory offers such an appropriate tool. In our game-theoretical framework, the state is playing against a pathogen by introducing non-pharmaceutical interventions to fulfil its socio-political goals, such as guaranteeing hospital care to all needed patients, keeping the country functioning, while the applied social restrictions should be as soft as possible. With the inclusion of activity and economic sector dependent transmission rate, optimal control of lockdowns and health care capacity management is calculated. We identify the presence and length of a pre-symptomatic infectious stage of the disease to have the greatest effect on the probability to cause a pandemic. Here we show that contrary to intuition, the state should not strive for the great expansion of its health care capacities even if its goal is to provide care for all requiring it and minimize the cost of lockdowns.

## Introduction

The COVID-19 pandemic once again called the attention to the serious effect the evolution of pathogens can have on contemporary societies. Evolution is not predictable as the generation of variation has an element of chance to it, so it cannot be exactly predicted when a novel, pandemic causing pathogen will emerge and with what kind of properties it will have. In order to prepare for an emerging infectious disease, the parameter dependence of a system involving both government measures and phenotypic parameters of the pathogen should be investigated. Furthermore, the socio-political objectives of the state also dictate the measures to take against the propagation of the pathogen.

Our aim is to introduce and study a conceptual, game-theoretical model that facilitates the preparedness of states to such emergencies. In this framework, first “Nature” (“Evolution”) moves (chooses strategy) in the sense that a so-far unknown pathogen, with properties allowing it to cause a pandemic emerges. Then it is a task of the state to work out an optimal strategy to handle the ensuing epidemic. The pathogen is such that (1) there is a non-ignorable presymptomatic period, when infected can transmit the pathogen to others^1–5^. This property combined with our current global travelling habits, make the quick outbreak of a pandemic possible^6^; (2) the death rate can be reduced by hospital care involving medical instruments, medicines and therapies already developed for other diseases^7^; (3) the death and hospitalization rate is not very high, thus the overwhelming majority of infected person does not need hospital care^7^; (4) the pathogen is transmitted mostly by person-to person contact, therefore the reduction of social contacts can slow down the propagation of the epidemic^8,9^. In the end, herd immunity will terminate the epidemic^10^, which can be reached through vaccination or by the majority recovering and becoming immune to the infection. If the propagation speed of the disease is high enough, the latter can dominate without the availability of sufficient amount of vaccine. Here we consider the worst-case scenario that vaccine is not developed before the epidemic runs its course.

The other player in case of a nation-wide epidemic is the state (government). We suppose that the state has two socio-political (shortly political) objectives: the first political objective is to guarantee hospital care for all citizens who require it^11^. We note that we will not consider the option that the state strive to save the maximum number of lives, since in that case it should maintain the most restrictive measures, until the sufficient amount of vaccine is at its disposal^12^. That solution would be economically^13^ and psychologically^14,15^ very costly. Second political objective is to minimize the restriction of liberty rights, i.e. minimize the extent and duration of lockdowns. The state has two ways to achieve its goals: (1) restrict social contacts to slow down the spread of the pathogen^16,17^, avoiding in this way the overload of the existing hospital capacity; and (2) increase hospital capacity, by which the duration of the epidemic can be shortened. We assume that the state always requires symptomatic individuals to quarantine themselves^18,19^, and it otherwise controls access to certain places and services.

**Figure 1:**
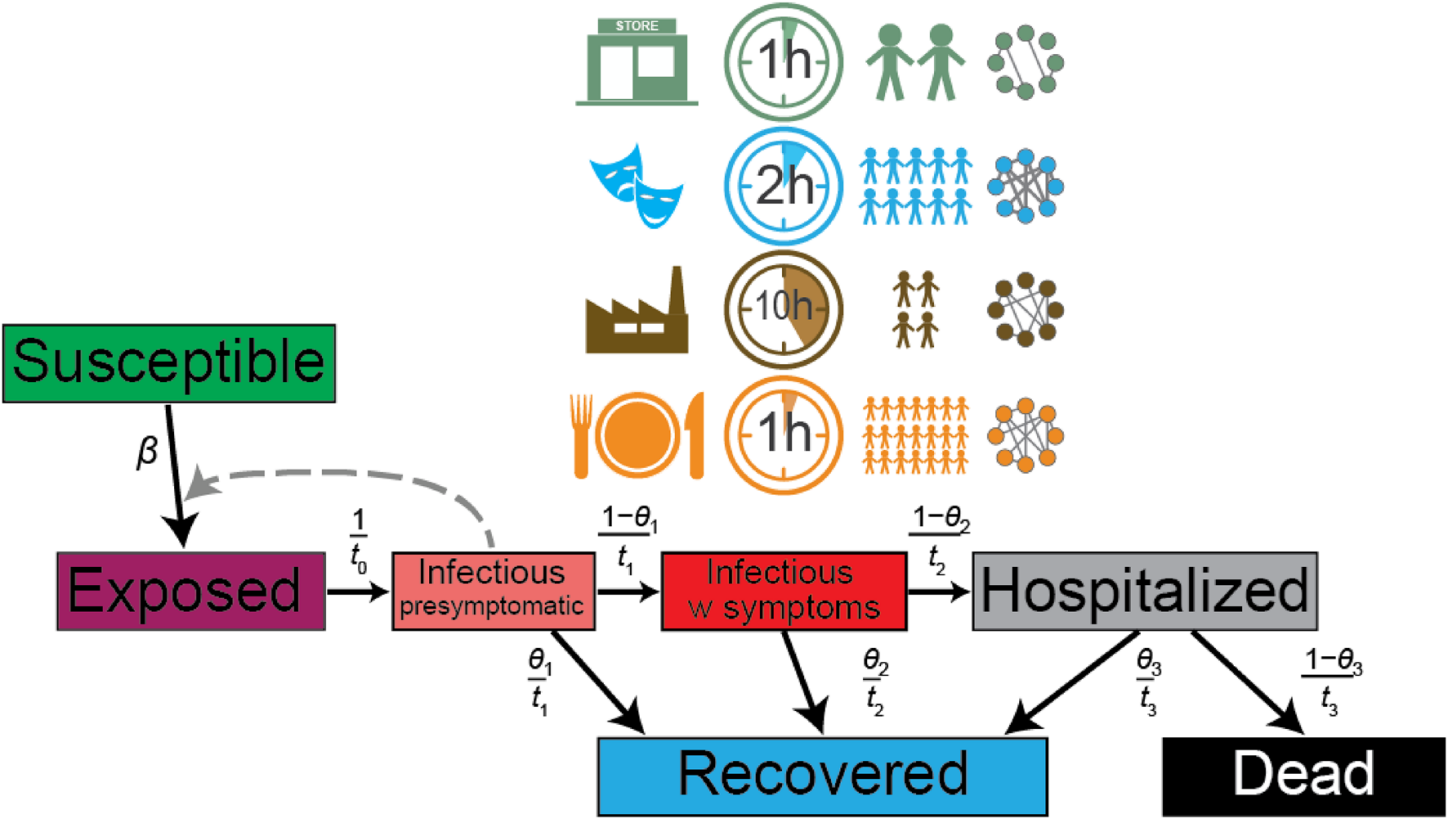
Schematic representation of the epidemiological model and the venue dependent transmission rate (probability). The modified SEIR epidemiological model has seven compartments. At the start, most individuals are susceptible. If infected, they become exposed, then proceed to infectious, but presymptomatic stage. Individuals can either recover at this stage or they can develop symptoms. Symptomatic infectious individuals either recover or their condition deteriorate, and they become hospitalized. Hospitalized individuals might be healed, or they pass away. Recovery from the corresponding state has a probability of $\theta_{i}$, whereas ${t}_{i}$ denotes the mean time of staying in the starting stage. Infectious individuals with symptoms are quarantined, and thus only infectious individuals without symptoms can infect others. Transmission probability (*β*) depends on the location which are characterized by the time people spend there, the mean density of people, their interaction intensity and connectivity. People, on average, spend most of their time in their workplace, which is a low density, low intensity and intermediate connectivity place. Stores have low density of people, who interact infrequently and with low intensity. On the other hand, entertainment and tourism are high density venues with high connectivity and intensity of interaction. See actual parameters in Supplementary Table 2.

Taking into account the above two objectives, using a modified SEIR epidemiological model with state control (Fig. 1), we investigate what the optimal non-pharmaceutical intervention strategy should be. The state optimizes its policies so that the short-term financial cost due to increased cost of health care and decrease of revenues from closed/restricted economic sectors is minimized. The payoff of the state in our game-theoretical model is the cost deduced revenues it has during the epidemic. Here we restrict our investigation to four sectors (retail stores; arts, entertainment and recreation; manufacturing; and travel, restaurants, hotels) with venues of these economic activities differing significantly in pathogen transmission potential^20,21^. We are fully aware that the economic consequences of a pandemic are more complex^22,23^, but our approach captures an important, oft-neglected characteristic, the time-dependent nature of the cost: longer lockdowns and more hospitalized patients result in higher cost for the entire country. The role of time constraints in evolutionary game theory^24–26^ and their inclusion in models of epidemic management^27^ is a new line of research.

## Method

### Conceptual model

We are modelling a game-theoretical situation between a state and an epidemic causing pathogen. Nature is considered to act first, i.e. a new pathogen has evolved, which can produce pandemic. A state wants to be prepared to the eventuality of such pandemic. The objective of the state during a pandemic is to realize its own social and healthcare politics, namely take targeted measures to ensure, with minimal social restrictions, hospital care to all seriously ill patients and sufficient number of active (asymptomatic) persons to run basic services. The state choses a target hospital capacity and optimizes a set of non-pharmaceutical interventions to reach the above objectives with the chosen hospital capacity. Both the cost of increased hospital capacity and the cost of economic restrictions decrease the payoff of the state. By the right choice of the number of beds (hospital capacity), the state can maximize its income during the epidemic.

The solution concept for our game model, in the context of this “game against Nature”, for Player 2 (state) it is to adopt Wald’s paradigm of *maximin* or *pessimistic solution* (also called conservative solution), see e.g.^54^: Player 2 counts with the “worst case”, supposing a strategy choice of Player 1 (pathogen) that minimizes the payoff of Player 2, and the latter maximizes this minimum. We emphasize that this maximin solution for Player 2 (state) is based on a unilateral approach. The actual strategy choice of the pathogen is not the focus of our study, we want to find the suitable strategy choice of the state. A pathogen does not need to take the worst strategy (from the point of view of the state) in a pandemic, but the state should be prepared for the worst case.

In order to set up our game-theoretical model, we have to take the following steps:

First, we introduce the dynamical model of the epidemic which is an extension of the SEIR epidemiological model. Here, transmission rates are derived for different types of venues which are places where different economic activities are conducted. These venues not only vary in their contributions to the economy (see below), but they also vary in crowding, length of average stays and other factors influencing the probability of catching the disease. This sub-model is important since it can handle the different shutdowns and their effect on the propagation of the pathogen. The restrictions influence the infection rate.

Second, we calculate the probability of a pathogen to cause a pandemic, i.e. to spread to other countries from its country of origin. If the pathogen does that, then countries all over the world need to deal with its consequences. Thus, the ability to cause a pandemic limits the strategy space of the pathogen. This sub-model will show us what kind of characteristics the focal pathogen in our game should have.

Third, we introduce the non-pharmaceutical interventions that are available to the state. These are the strategic options available to the state against the epidemic caused by a novel pathogen. The state implements optimized control on access to various services, economic activities, etc. in order to curb the spread of the infection so that the hospital capacities are never overextended but at the same time the least amount of restrictions are implemented. The state control guarantees that it reaches its socio-political objectives.

Fourth, we formally introduce the game against Nature considering different possible novel pathogens. In our game, the novel pathogen is characterized by the time infected individuals stay on average in the presymptomatic, infectious stage. We will consider four types of pathogens and four increased hospital capacities, calculating the payoff of the state as the net income under the control implying that the socio-political objectives are reached. Under the constraint of its political goal, the state maximizes its net income during the epidemic, considering the worst-case strategy choice of the pathogen.

### General epidemiological model

We employ an extended version of the SEIR compartment model of epidemiology: apart from the standard Susceptible, Exposed and Recovered stages, we explicitly count the Deceased, and divide the Infectious phase into three subphases: presymptomatic infectious, symptomatic infectious and seriously ill infectious. Susceptible individuals ($S$) can become infected if they meet infectious ($I_{{{\text{ps}}}}$, $I_{{\text{s}}}$, $I_{{\text{h}}}$) individuals. Infected individuals ($E$) enter the Exposed stage, and after the incubation period ($t_{0}$) they enter the Infectious (presymptomatic) stage. Infectious individuals either recover with transition probability $\theta_{i}$ (depending on the stage) or advance to the next Infectious stage (presymptomatic → symptomatic → seriously ill/hospitalized) with characteristic times $t_{1}$, $t_{2}$ and $t_{3}$, respectively. The rate of transitions from one stage to the next in these cases depend on the probability of recovery $\theta_{i}$ ($i \in \left\{ {1,2,3} \right\}$) and the average time, $t_{i}$ time ($i \in \left\{ {1,2,3} \right\}$), spent in the given stage. Individuals either recover with rate $\frac{{\theta_{i} }}{{t_{i} }}$ or after spending, on average, $t_{i}$ time in the current stage before transitioning to the next stage. Consequently, the rate of transition is $\frac{{1 - \theta_{i} }}{{t_{i} }}$. Individuals who do not recover from the seriously ill condition die (enter the Deceased compartment). Seriously ill individuals can only recover if they get medical care, without medical care these individuals would die. We assume that such care is available, even though it is not guaranteed that there is a treatment that can help people with the complications arising from a novel pathogen. Recovered individuals ($R$) remain immune to the infection i.e. the acquired resistance is long lasting, at least till the calm down of the epidemic, in other words, the recovered patients do not get infected again. Table 1 lists all variables and parameters of the model.

**Table 1:** Variables and parameters of the model (times in days).

Variable	Meaning	Employed values
${t}_{0}$	time for non-infectious non-symptomatic incubation period	5
${t}_{1}$	time for infectious, but non symptomatic incubation period	1.393; 2.3217; 3.2504; 4.1791
${\theta }_{1}$	probability of going to the recovered state from the non-symptomatic infectious stage	0.3
${t}_{2}$	time for being a symptomatic, infectious individual	8
${\theta }_{2}$	probability of going to the recovered state from the symptomatic infectious stage without requiring hospitalization	0.8
${t}_{3}$	the mean time seriously ill individuals are hospitalized	14
${\theta }_{3}$	probability of surviving hospitalization	0.9
${R}_{\mathrm{E}}$	effective reproductive number	1.5; 2.5; 3.5 and 4.5

With the above notations, the death rate of an infected individual without available treatment is $\delta_{0} = \mathop \prod \limits_{i = 1}^{2} \left( {1 - \theta_{i} } \right)$, and it is $\delta_{1} = \mathop \prod \limits_{i = 1}^{3} \left( {1 - \theta_{i} } \right)$ if hospitalization is available.

The following differential equations govern the dynamics:

$$\begin{aligned} \frac{{dS}}{{dt}} & = - \beta S \\ \frac{{dE}}{{dt}} & = \beta S - \frac{1}{{t_{0} }}E \\ \frac{{dI_{{{\text{ps}}}} }}{{dt}} & = \frac{1}{{t_{0} }}E - \frac{{1 - \theta _{1} }}{{t_{1} }}I_{{{\text{ps}}}} - \frac{{\theta _{1} }}{{t_{1} }}I_{{{\text{ps}}}} = \frac{1}{{t_{0} }}E - \frac{1}{{t_{1} }}I_{{{\text{ps}}}} \\ \frac{{dI_{{\text{s}}} }}{{dt}} & = \frac{{1 - \theta _{1} }}{{t_{1} }}I_{{{\text{ps}}}} - \frac{{1 - \theta _{2} }}{{t_{2} }}I_{{\text{s}}} - \frac{{\theta _{2} }}{{t_{2} }}I_{{\text{s}}} = \frac{{1 - \theta _{1} }}{{t_{1} }}I_{{{\text{ps}}}} - \frac{1}{{t_{2} }}I_{{\text{s}}} \\ \frac{{dI_{{\text{h}}} }}{{dt}} & = \frac{{1 - \theta _{2} }}{{t_{2} }}I_{{\text{s}}} - \frac{{\theta _{3} }}{{t_{3} }}I_{{\text{h}}} - \frac{{1 - \theta _{3} }}{{t_{3} }}I_{{\text{h}}} = \frac{{1 - \theta _{2} }}{{t_{2} }}I_{{\text{s}}} - \frac{1}{{t_{3} }}I_{{\text{h}}} \\ \frac{{dR}}{{dt}} & = \frac{{\theta _{1} }}{{t_{1} }}I_{{{\text{ps}}}} + \frac{{\theta _{2} }}{{t_{2} }}I_{{\text{s}}} + \frac{{\theta _{3} }}{{t_{3} }}I_{{\text{h}}} \\ \frac{{dD}}{{dt}} & = \frac{{1 - \theta _{3} }}{{t_{3} }}I_{{\text{h}}} . \\ \end{aligned}$$

Here the transmission rate is

1 $$\beta = 1 - \mathop \prod \limits_{i = 1}^{4} \left( {1 - \alpha_{i} \left( {1 - \left( {\frac{S + E + R}{{S + E + I_{{{\text{ps}}}} + I_{{\text{s}}} + R}}} \right)^{{k_{i} }} } \right)} \right)^{{\tau_{i} }},$$

where

- $k_{i}$ denotes the *interaction intensity:* A focal individual in venue $i$ (place of economic activity) is supposed to interact with $k_{i}$ people during one time period (15 min in our case). In other words, $k_{i}$ is the maximal number of susceptible people to whom the disease can be transmitted by an infected individual in one time period (handshake, being in a distance of less than two meters, sneezing, etc.)
- ${\alpha }_{i}$ denotes the probability of a susceptible person to get infected if exposed the infection, in venue $i$, during one time period (15 min in our case). This depends on the size of the indoor place, ventilation, air flows, etc. It does not depend on the number of infectious persons (provided it is not zero) among the ${k}_{i}$ people in the neighbourhood of the focal individual (e.g., if the disease is highly contagious, the exposure to infection is high even for a single infectious interaction).
- ${\tau }_{i}$ denotes the number of time periods a person spends on average in venue $i$ during one day (the length of one period is 15 min in our case). Sometimes it is called the repetition number.

The derivation of Eq. (1) is detailed in the Supplementary Information, Section 3.

Another important epidemiological characteristic is the basic reproductive number ${R}_{0}$. As the viral load can be different at different stages of the infection, we calculate the basic reproductive number by the following equation:

$$R_{0} = r_{1} t_{1} + \left( {1 - \theta_{1} } \right)r_{2} t_{2} + \left( {1 - \theta_{1} } \right)\left( {1 - \theta_{2} } \right)r_{3} t_{3},$$

where $r_{i}$ is the mean number of individuals infected by a stage $i$. The effective reproductive number ($R_{{\text{E}}}$) is a similar quantity with the assumed quarantining of sick (symptomatic) individuals, but without any other non-pharmaceutical intervention in place (see later),

$$R_{{\text{E}}} = r_{1} t_{1} .$$

Initially we assume that the whole population is susceptible to the new pathogen, thus everyone starts in the susceptible compartment. In each case of our investigation, the epidemic starts with 100 infected individuals.

We are aware of the fact the progress of a disease cannot be described as a Markov process as the probability of advancing to the next stage increases with the time spent in the current stage. The time spent in the stages can be approximated with lognormal, Weibull and gamma distributions^28^. If we model great many individuals, the fraction of people leaving the given stage can adequately be approximated with the inverse of the expected (mean) time spent in the given stage.

This extended SEIR compartment model is employed when determining the probability of a pathogen causing a pandemic. If a pandemic unfolds, the states implement non-pharmaceutical interventions which, as assumed here, preclude certain individuals to infect others as they are quarantined. When calculating optimal control, a slightly modified set of equation is used as described below.

### Activity dependent basic reproduction number

Our model makes it possible to introduce an *activity dependent reproduction number* ${R}_{i}^{*}$ for each venue type $i$. This is the average number of secondary infections caused by single infectious individual provided she/he stays in venue $i$ during the whole infectious period ${t}_{1}$ and everybody else is susceptible. Such index numbers are useful in modelling the initial phase of the outbreak when the epidemic is still not recognized, and also in studying of the effects of restrictions. This is what we applied when determining the transmission rate ${r}_{1}$ for the model introduced above.

For the sake of simplicity, we now suppose that there are ${n}_{i}$ susceptible people in venue $i$ at every moment (in addition to the single infectious individual). We suppose that there are ${k}_{i}$ people within the range of infection, and considering an otherwise well-mixed population, the probability of a given susceptible individual to be at risk is $k_{i} /n_{i}$. As the number of repetitions is $\tau_{i}$, and the chance of acquiring infection is $\alpha_{i}$ at each occasion, every susceptible individual has probability

$$p_{i}^{*} = \left( {1 - \alpha_{i} \frac{{k_{i} }}{{n_{i} }}} \right)^{{\tau_{i} }}$$

of getting off. Hence, the probability of getting ill is $\beta_{i}^{*} = 1 - p_{i}^{*}$. Note that these quantities are not equal to $p_{i}$ and $\beta_{i}$ computed above. The difference lies in the points of view: $\beta_{i}$ is the probability of a *susceptible* individual to get infected in venue $i$, while $\beta_{i}^{*}$ is the probability that a single *infectious* individual transmits the disease to a fixed susceptible person in venue $i$.

How many susceptible people does an infectious individual meet during the infectious period? Let $c$ be the rate of conversion between time (measured by the day) and repetition periods, that is, one repetition period is equal to $c$ times of a day. For example, if one repetition period is 15 min, then $c = \frac{1}{4 \times 24}$. Then the time a susceptible person spends in venue $i$ is $c\tau_{i}$ (on average, measured in days). Thus, during the whole infectious period

$$\frac{{n_{i} t_{1} }}{{{ }c\tau_{i} }}$$

susceptible persons get close to the source of infection. Again, for the sake of simplicity, we suppose that they are all different, though it is not necessarily true: think of the regulars in a pub. This simplification may lead to the overestimation of the number of secondary infections.

All in all, the average number of secondary infections in venue $i$ is

$$R_{i}^{*} = \frac{{n_{i} t_{1} }}{{c\tau_{i} }}\beta_{i}^{*} = \frac{{n_{i} t_{1} }}{{c\tau_{i} }}\left[ {1 - \left( {1 - \alpha_{i} \frac{{k_{i} }}{{n_{i} }}} \right)^{{\tau_{i} }} } \right] \approx c^{ - 1} k_{i} t_{1} \alpha_{i} .$$

For the approximation in the right-hand side a small value of $\alpha_{i} \frac{{k_{i} }}{{n_{i} }}$ should be assumed. E.g., the radius of infection is small, and/or the probability $\alpha_{i}$ of transmitting the disease by a single encounter is small.

Note that all these considerations only apply to venues different from one’s home: there $k_{0} = n_{0}$, the average number of relatives the infectious individual shares her/his residence, and the venue specific reproduction number would be $n_{0} \left[ {1 - \left( {1 - \alpha_{0} } \right)^{{t_{1} /c}} } \right]$. This is misleading, however, because relatives infected at home cannot infect *further* relatives at the same rate, only each other, consequently not spreading the disease.

Let $r_{1}$ denote the expected number of cases directly generated by one case being infectious without symptoms, in a population where all individuals are susceptible to infection, during one day. What is the connection between the transmission rate $r_{1}$ and the $i$-venue reproduction numbers $R_{i}^{*}$ introduced above? Let’s assume, there are $H$ different venues. Family members living together can acquire each other’s infection more easily, but, especially during social distancing measures, not every member, typically just one, of the family leaves home for shopping etc. Thus, households can be considered as single units/individuals regarding the spread of the epidemic, hence transmitting infection at one’s home may be left out of consideration. Consequently, the overall transmission rate in the non-symptomatic infectious stage is

2 $$r_{1} = \mathop \sum \limits_{i = 1}^{H} n_{i} \beta_{i}^{*} = \mathop \sum \limits_{i = 1}^{H} n_{i} \left[ {1 - \left( {1 - \alpha_{i} \frac{{k_{i} }}{{n_{i} }}} \right)^{{\tau_{i} }} } \right],$$

thus

$$t_{1} r_{1} = \mathop \sum \limits_{i = 1}^{H} w_{i} R_{i}^{*}, {\text{where }} w_{i} = \frac{{\tau_{i} }}{{\tau_{0} + \mathop \sum \nolimits_{i = 1}^{H} \tau_{i} }} .$$

Note that $c\left( {\tau_{0} + \mathop \sum \limits_{i = 1}^{H} \tau_{i} } \right) = 1$, because the total time an individual spends on the different venues per day is just the whole day. Thus, $w_{i} = c\tau_{i}$; this is the proportion of time an infectious individual spends in venue $i$, and

$$t_{1} r_{1} = c\mathop \sum \limits_{i = 1}^{H} \tau_{i} R_{i}^{*} .$$

### Estimation of the chance of a pandemic outbreak by taking into account of time constraints and recovery rates

A pandemic emerges almost certainly if the mutant pathogen can spread into other countries before the new epidemic is identified. Countries need to be prepared for such pathogens. Thus, the characteristics of such pathogens guide us in the parameters to use as the strategy option for the pathogen.

Ample travel connections between countries allow the escalation of the pandemic^6^. Let $U_{C}$ stands for the traveler pool going out of a country with population size *N*. In a well-mixed model, the probability that an individual will travel to a foreign land is $U_{C} /N$. In a case of a novel pathogen, when the symptoms of the disease it causes has not yet been determined, there could be no quarantine, thus both pre-symptomatic infectious (*I*_ps_) and symptomatic infectious individuals (*I*_s_) can infect others, but we can assume that the seriously ill (*I*_h_) do not travel.

Now we examine this problem from the aspect of time constraints. The following two time periods play important roles in the pandemic outbreak.

*Detection time*: It takes time to realize that a newly emerged pathogen is causing disease. This detection time can depend on several factors (e.g. the developmental state of the health care system, the openness of communication, etc.). This is one of the parameters of our investigation denoted by $T_{C}$, which can vary between 0 and 2 months ($T_{C} \in \left( {0,2} \right)$).

*Incubation time*: A necessary condition for a pandemic is that during time $T_{c}$ an infected individual (*E*, *I*_ps_, *I*_s_) travels aboard, i.e. enters the traveller pool ($U_{C}$).

Branching processes can be used to investigate the emergence of epidemics^6^. As the probability of recovery ($\theta_{i}$) and the mean time ($t_{i}$) spent in given stage is different at different stages of the infection, we employ a general, time-dependent branching process (Crump–Mode–Jagers process) (cf.^29,30^) to investigate the risk of new pathogen outbreaks in countries outside of the country of origin.

We emphasize here again, that at the start of the epidemic, the pathogen is not yet identified and consequently, infectious symptomatic individuals are allowed to travel. We also assume that presymptomatic stage is considerably shorter than the time considered for the identification of the new pathogen. Pathogens with extremely long incubation periods are not considered here as in nowadays society, almost everybody would get infected before any preventive measure could be initiated.

The spread of an epidemic in the early stage can be modelled with a general time-dependent branching process (Crump–Mode–Jagers process, see ^31^ or ^32^ for the exact but still not too technical definition and basic properties). In the terminology of such processes, the individuals of the process are the infected people, production of offspring is the transmission of the disease, and lifetime means the length of the infectious period, ending with recovery or getting quarantined or hospitalized.

**Figure 2:**
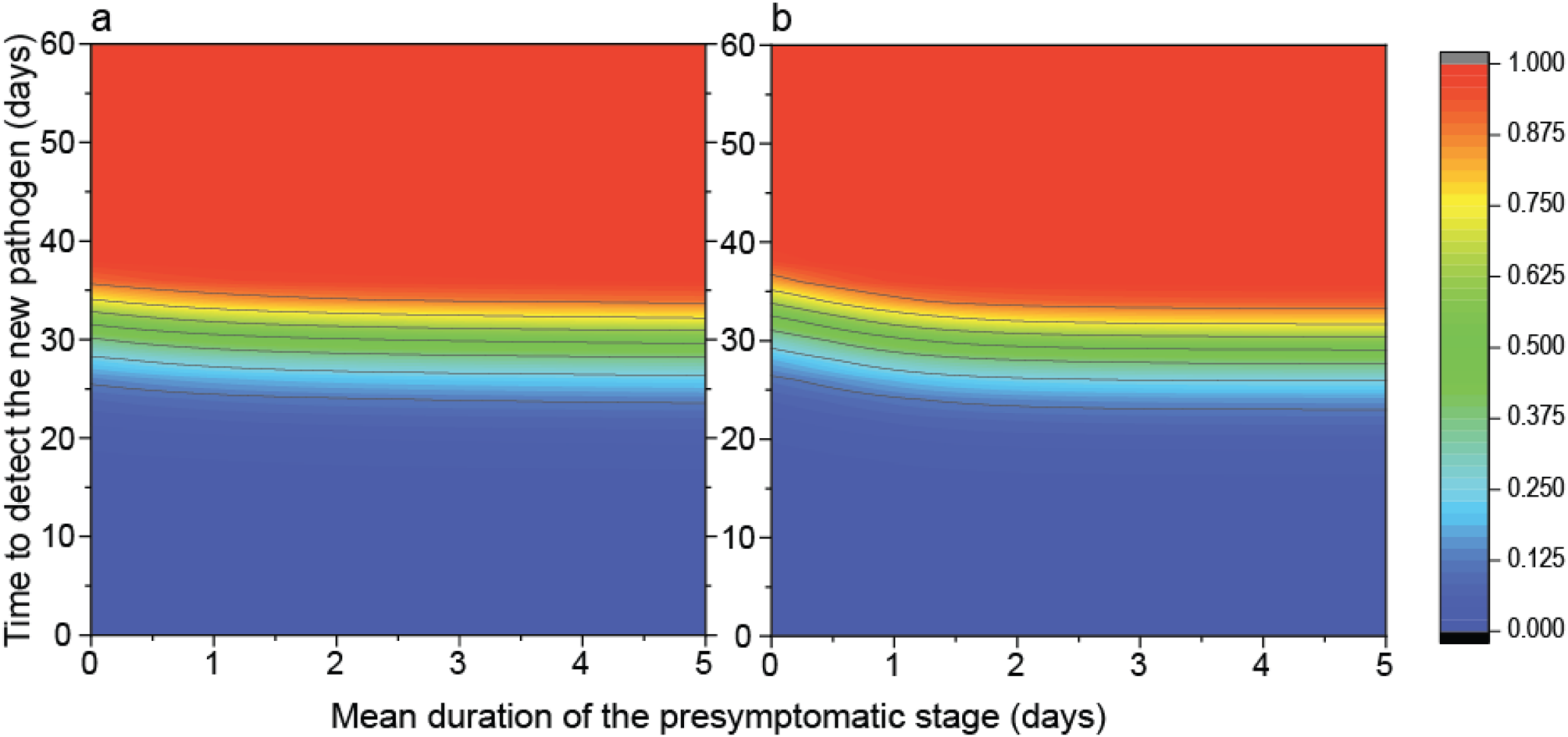
Probability of a pandemic as function of time to detect the new pathogen (Y axes) and the mean duration of the presymptomatic stage (X axes). We illustrate our analytical result on the probability of an epidemic outflowing to other countries, thus causing a pandemic. Probabilities are calculated according to the formula given in Supplementary Equation (SI.2). The recovery rate from the presymptomatic infectious stage is (**a**) $\theta_1 = 0.3$; and (**b**) $\theta_1 = 0.6$. Other parameters are $t_0 = 5$, $t_2 = 8$ (days), $U_C /N = 0.00015$, $r_1 = 1.07678$.

The in-depth formalization and solution of the time-dependent branching process is detailed in the Supplementary Information, section 2. In this way we can compute the spreading probability in our model. On Fig. 2 this probability is plotted against $t_{1}$ and $T_{C}$, with the following parameters: $T_{C} \in \left[ {0, 60} \right]$, $t_{1} \in \left[ {0, 5} \right]$, $t_{0} = 5$, $t_{2} = 8$ (days), $U_{C} /N = 0.00015$, $r_{1} = 1.07681$ (computed according to Supplementary Table 2, based on Eq. (2)), and $r_{2} = 2r_{1}$. Two values of $\theta_{1}$ are considered: $\theta_{1} = 0.3$ (Fig. 2a) and $\theta_{1} = 0.6$ (Fig. 2b).

### State-optimally controlled epidemic model

We construe the fight against an epidemic by the state as a game-theoretical situation between a novel pathogen (Player 1) and a state (Player 2). Player 1’s strategy is the characteristic of the pathogen. As we have seen above ${t}_{1}$, the time period of pre- or non-symptomatic infectious period is key to cause a pandemic, consequently strategy of Player 1 is ${t}_{1}$. Once a pathogen reaches a country, and the extent of the infection cannot be controlled by contact tracing, then other measures need to be taken, otherwise the number of hospitalized (${I}_{\mathrm{h}}$) patients will exceed the hospital capacity. Consequently, the state can mandate certain non-pharmaceutical interventions to curb the epidemic.

- *Quarantining of symptomatic individuals.* Individuals showing symptoms are immediately quarantined at home (with mild symptoms) or in hospitals if seriously or critically ill. Consequently, symptomatic (${I}_{\mathrm{s}}$) and seriously ill (${I}_{\mathrm{h}}$) infectious individuals cannot infect others. The time lag between the onset of symptoms and actual quarantining is an important factor in containing a pandemic^33^. The quarantine is assumed to be perfect, citizens obey it for the greater good or the state has the means to enforce it. While without quarantine, symptomatic infectious individuals can be more infectious than asymptomatic infectious individuals^38–40^ (there is a modelling study assuming that they are twice as infectious^41,42^), if they cannot meet susceptible individuals, then they cannot transmit the infection. These measures are in effect throughout the epidemic irrespective of the actual extent of the infection. Taking the above together, the infection can only spread via those in the pre-symptomatic infectious state (*I*_ps_).
- *No nosocomial infection.* We assume that individuals in the hospitals cannot infect new individuals, as they are sealed off from the outside world. Doctors, nurses and others working with COVID-19 patient are also separated from the rest of the populace to minimize the effect of nosocomial infections. Early in a pandemic, hospital acquired infection and infection among the medical staff could be high^34,35^. However, with precautions, such as universal mask wearing, widespread testing, no visitor policy, negative pressure rooms and adequate personal protective equipment, hospital acquired infection can be prevented^36,37^.
- *Members of a household are quarantined together.* We also assume there is no further separation of infected and non-infected within a household. We assume that ${t}_{0}$ is greater than ${t}_{1}$, so household members infected will show symptoms before the quarantined is lifted, so they won’t be spreading the disease in the greater population. This assumption is valid for COVID-19, but not for influenza. Consequently, household transmission does not affect the spread of the disease.
- *Installation of increased hospital capacity.* The state can increase its initial hospital capacity ($Y_{0}$) to a higher value ($Y_{3}$) ($Y_{3} \ge Y_{0}$) which is enough to care for all seriously ill during the epidemic. The choice of higher hospital capacity means that the state (Player 2) has to establish $Y_{3} - Y_{0}$ new hospital bed capacity, at cost $h_{0}$ each, and cover the daily cost $h_{1}$ of a hospital bed in service.
- *Venue based access restrictions.* The state can further mitigate the spread of the epidemic by selectively allowing or forbidding certain economic activities. Forbidding these activities, for example closing shops, cinemas, hotels or prohibiting gatherings, like concerts, decrease contacts among citizens and thus lowers the probability of transmission. We have chosen four board categories of venues, ones that were chosen to be closed in some European countries during the COVID-19 pandemic in the spring of 2020. These venues represent^1^ arts, entertainment and recreation;^2^ tourism;^3^ retails shops, excluding those selling food, hygiene products and pharmacies; and^4^ manufacturing excluding those producing essential products. As the transmission rate can be different in these places and the economic cost of their lockdown can be different, the state could implement a mix of restrictions that would satisfy its societal and economic goals. The state updates the mix of restrictions on a weekly basis. While technically any discrete time-interval could be used, a week is a compromise between fine-scaled control and still allowing the actors of the economy to respond. Let $u$ describe the restrictions (with $u_{i} = 1$ meaning no restriction, and $u_{i} = 0$ full ban). Suppose that normally, the total yearly contribution of the activity, sector or venue $i$ to the GDP is $g_{i}$, which is then lowered when restrictions apply to them. This is another cost for Player 2. Consequently Player 2 solves the optimal control problem (see below) given Player 1’s strategy $t_{1}$ and its number of hospital beds, $Y_{3}$. Let $u^{*}$ be the corresponding optimal control.

With the above non-pharmaceutical interventions, the general epidemiological model is modified to that shown in Fig. 1.

3 $$\frac{dS}{{dt}} = - \beta S$$

4 $$\frac{dE}{{dt}} = \beta S - \frac{1}{{t_{0} }}E$$

5 $$\frac{{dI_{{{\text{ps}}}} }}{dt} = \frac{1}{{t_{0} }}E - \frac{1}{{t_{1} }}I_{{{\text{ps}}}}$$

6 $$\frac{{dI_{{\text{s}}} }}{dt} = \frac{{1 - \theta_{1} }}{{t_{1} }}I_{{{\text{ps}}}} - \frac{1}{{t_{2} }}I_{{\text{s}}}$$

7 $$\frac{{dI_{{\text{h}}} }}{dt} = \frac{{1 - \theta_{2} }}{{t_{2} }}I_{{\text{s}}} - \frac{1}{{t_{3} }}I_{{\text{h}}}$$

8 $$\frac{dR}{{dt}} = \frac{{\theta_{1} }}{{t_{1} }}I_{{{\text{ps}}}} + \frac{{\theta_{2} }}{{t_{2} }}I_{{\text{s}}} + \frac{{\theta_{3} }}{{t_{3} }}I_{{\text{h}}}$$

Here the transmission rate is

$$\beta = 1 - \mathop \prod \limits_{i = 1}^{4} \left( {1 - \alpha_{i} \left( {1 - \left( {\frac{S + E + R}{{S + E + I_{{{\text{ps}}}} + R}}} \right)^{{k_{i} }} } \right)} \right)^{{\tau_{i} }} .$$

This can be computed in the same way as we did for the general epidemiological model. The only difference is that infected people with symptoms are now prevented from transmitting the disease, hence their number $I_{{\text{s}}}$ is missing from the denominator.

### The formal description of the optimal control model

It is intuitively obvious that by appropriate restrictions *u*_*i*_ it can be achieved that all involved citizens receive the necessary medical care. To this end, the following optimal control problem is solved.

The controlling measures can be described by a vector $u = \left( {u_{1}, \ldots, u_{H} } \right)$, where $u_{i} \in \left[ {0, 1} \right]$. Here $u_{i}$ shows how small a proportion the time spent in or access to venue $i$ is reduced to, due non-pharmaceutical interventions implemented by the state. For example, if $u_{1} = \ldots = u_{H} = 1$, then there are no restrictions at all. On the other hand, $u_{i} = 0$ means the total prohibition of the given activity. We suppose that the plus time saved by the controlling measures is spent at home. In this way

$$\beta \left( u \right) = 1 - \mathop \prod \limits_{i = 1}^{H} \left( {1 - \alpha_{i} \left( {1 - p_{i} } \right)} \right)^{{u_{i} \tau_{i} }} .$$

Particularly, in our case this is

9 $$\beta \left( u \right) = 1 - \mathop \prod \limits_{i = 1}^{4} \left( {1 - \alpha_{i} \left( {1 - \left( {\frac{S + E + R}{{S + E + I_{{{\text{ps}}}} + R}}} \right)^{{k_{i} }} } \right)} \right)^{{u_{i} \tau_{i} }} .$$

The risk of infection is obviously minimal in the case of total prohibition. Then everybody is in quarantine and only basic needs are provided. (There is a special intervention for basic services, corresponding to $i = 1$ (supermarkets, pharmacies etc.) where the activity cannot be reduced to zero.) If $u_{1}^{0}$ denotes the admissible minimal level of essential services for providing basic needs, then

$$\mathop {\min }\limits_{u} \beta \left( u \right) = 1 - \left( {1 - \alpha_{1} \left( {1 - p_{1} } \right)} \right)^{{u_{1}^{0} \tau_{1} }} = 1 - \left( {1 - \beta_{1} } \right)^{{u_{1}^{0} }} .$$

However, such a strict control might prolong the epidemic and destroy the economy. In fact, the control vector $u$ varies with time, thus it is a function $u\left( t \right)$ rather than a constant vector. Introducing constraints and a suitable objective function, we arrive at a dynamic control problem.

*Class of admissible controls*: Fix a time interval [0,*T*], and define the set *U*[0,*T*] of admissible controls *u*(*t*) = (*u*_1_(*t*), *u*_2_(*t*), *u*_3_(*t*), *u*_4_(*t*)), where *u*_*i*_ are step functions defined on time interval [0,*T*], with base intervals of length 7 days (a week), and $u_{i} \left( t \right) \in \left[ {0,1} \right]$ $\left( {t \in \left[ {0,T} \right]} \right)$. The measures taken by the state influence the probability that an infection will happen in the *i*-th venue category, in the form $u_{i} \left( t \right)\tau_{i}$. Furthermore, there is a special intervention for basic services, corresponding to *i* = 1 (including basic food supply and pharmacies), where the activity cannot be reduced to zero, so $u_{1} \left( t \right) \in \left[ {u_{1}^{0} ,1} \right]$ with some $u_{1}^{0} > 0$. The dynamics of the control problem is given by the epidemiological model (3)-(8), with control-dependent transmission rate (9).

Now the optimal control problem that would express certain political aims of the government, is

10 $$\mathop \sum \limits_{i = 1}^{4} \mathop \smallint \limits_{0}^{T} u_{i} \left( t \right)dt \to max,\;(u \in U\left[ {0,T} \right]),$$

1. Keep the necessary hospital care under a given capacity $Y_{3}$:
  11 $$I_{{\text{h}}} \left( t \right) \le Y_{3 }\; \left( {t \in \left[ {0,T} \right]} \right),$$
  and
2. Keep the number of active citizens above a threshold *M*, to keep society working:
  12 $$S\left( t \right) + E\left( t \right) + I_{{{\text{ps}}}} \left( t \right) + R\left( t \right) \ge M\;\;\left( {t \in \left[ {0,T} \right]} \right).$$

We note that the larger the integral (Eq. 5) is, the lighter the restrictions are.

Observe that the maximization of objective functional (Eq. 10) corresponds to the socio- political objective that the social restrictions should be as soft as possible (i.e. the controls $u_{i} \left( t \right)$ should be as large as possible). Constraint (Eq. 11) ensures that there are enough hospital beds for all $I_{{\text{h}}}$-stage patients. Constraint (Eq. 12) guarantees that there are sufficient asymptomatic persons to maintain all basic functions of the state. In the definition of the payoff of the game model, the solution $\left( {u_{1}^{*} \left( t \right), u_{2}^{*} \left( t \right),u_{3}^{*} \left( t \right),u_{4}^{*} \left( t \right)} \right)$ of this optimal control problem is used, see (Eq. 13) of the next section, where economic issues are also involved.

The solution of the optimal control problem was programmed in MATLAB environment, see ^43,44^. We employed the standard solver found in MATLAB.

### The formal description of the game-theoretical model

The strategy of the pathogen is ${t}_{1}$ the mean time of the presymptomatic infectious state and the strategy of the state is level of extra hospital capacity it installs (${Y}_{3}$). The payoff to Player 2 is defined as the difference between the total income and total costs, for the whole duration of the epidemic, corresponding to strategy pair $\left( {t_{1},Y_{3} } \right)$:

13 $$f\left( {t_{1},Y_{3} } \right) = \mathop \sum \limits_{i = 1}^{4} \frac{{g_{i} }}{365}\mathop \smallint \limits_{0}^{{T_{1} }} u_{i}^{*} \left( t \right)dt + \left( {T_{3} - T_{1} } \right)\mathop \sum \limits_{i = 1}^{4} \frac{{g_{i} }}{365} - h_{0} \left( {Y_{3} - Y_{0} } \right) - h_{1} \mathop \smallint \limits_{0}^{{T_{3} }} I_{{\text{h}}} \left( t \right)dt.$$

Here $g_{i}$ is the gross value added for venue (sector) *i*. The first term thus measures the income of the state modified by the control, i.e. if there are extensive lockdowns and restriction then there is less income from those economic activities. To calculate the payoff, for every fixed pair $\left( {t_{1},Y_{3} } \right)$, we need the optimal controls obtained from the numerical investigation (see examples in Figs. 4 and 5 of the next section).

From the economic point of view, the epidemic lasts for the duration *T*_1*,*_ (Supplementary Table 5) after which all controls $u_{i}$ equal 1, i.e. there are no restrictions. On the other hand, from a public health perspective, the epidemic ends at time *T*_3_ (Supplementary Table 6), when the number of hospitalized patients decreases below $Y_{3} /1000.$ During this time ($T_{3} - T_{1}$) the economy is fully functional, but there could still be extra cost as patients are hospitalized. This is the second term in the equation. The first two terms are the income for the state.

The third term is the cost of new hospital beds ($Y_{3} - Y_{0}$), and the fourth term is the cost of hospitalizations. $I_{h} \left( t \right)$ is the number of patients in hospital at time *t*; and $C = \mathop \smallint \limits_{0}^{{T_{3} }} I_{h} \left( t \right)dt$ is the total number of hospital days during time $T_{3}$ (Supplementary Table 7).

The solution for the game is as follows. Considering the values $f\left( {t_{1},Y_{3} } \right)$ as entries of a corresponding 4 × 4 payoff matrix $M = \left[ {m_{jk} } \right],$ formally, $k_{0}$ is a *maximin solution for Player 2*, if

$$\mathop {\max }\limits_{k} \mathop {\min }\limits_{j} m_{jk} = \mathop {\min }\limits_{j} m_{{jk_{0} }}.$$

## Results

A pandemic will unfold if it takes too long to identify the pathogen or if infectious individuals are unaware of their condition for a long time because they are not symptomatic. Pathogens having characteristics that allow them to cause pandemics are the ones states need to be prepared for. If we parametrize the SEIR model so that it resembles the parameters of the COVID-19 epidemic (see Supplementary Methods), then we predict that the epidemic will spread into other countries with high probability, and thus causing a pandemic, if the identification needs more than two months (Fig. 2). In such case, the probability of the pandemic does not depend on the duration of the presymptomatic stage. On the other hand, if the pathogen gets identified in about 30 days, then the average duration of the presymptomatic stage plays an important role: a higher value can significantly increase the chance of a pandemic. Consequently, the duration of the presymptomatic stage is considered to be the strategy of the pathogen. We assume that the pandemic has developed, and states need to face this challenge.

**Figure 3:**
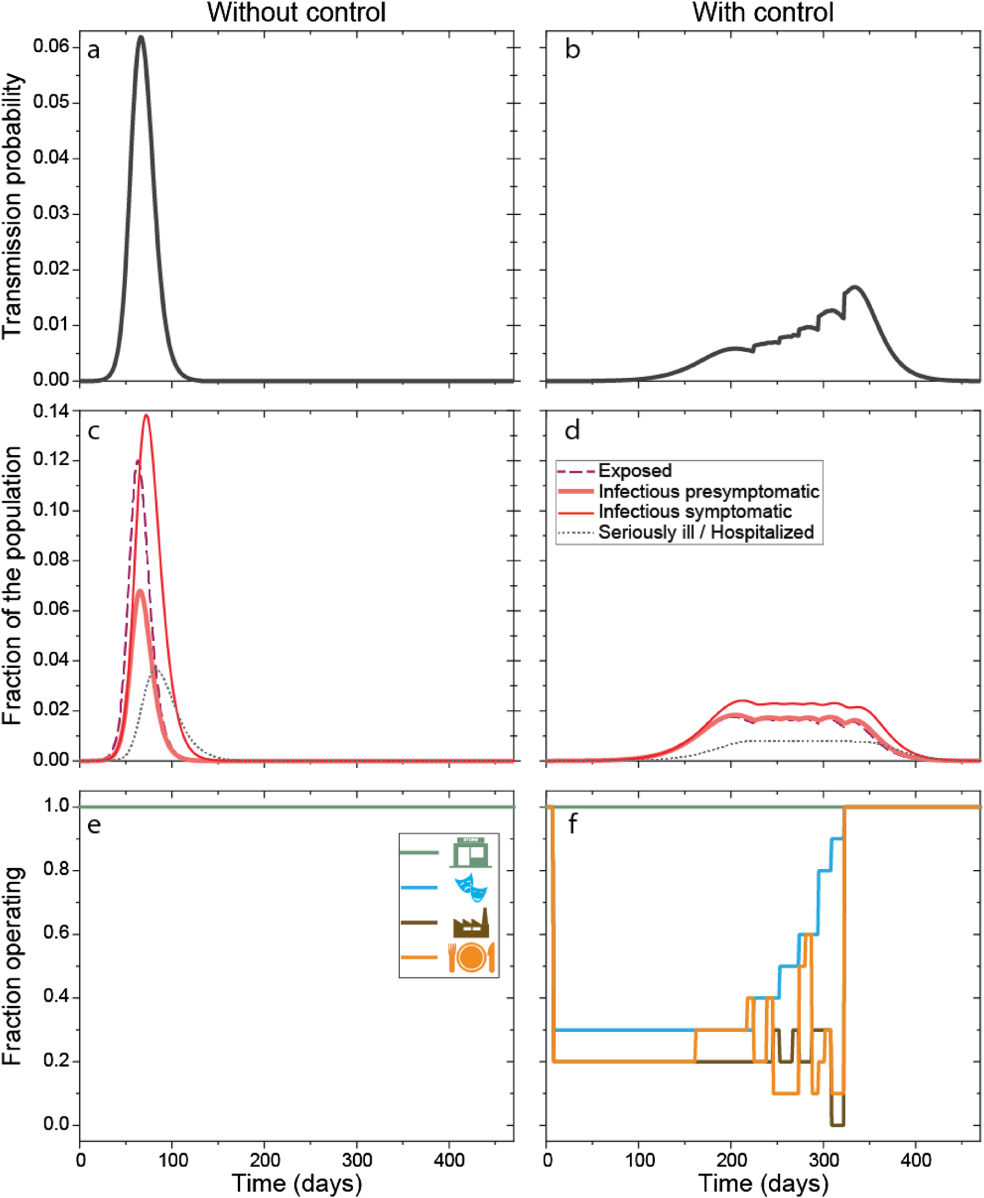
Comparison of the course of the epidemic without control (**a**, **c**, **e**) and with control (**b**, **d**, **f**). Transmission probability (**a**, **b**) can be controlled by the selective (partial) closing or opening of certain venues (economic sectors) (**e**, **f**), which leads to a longer (compare c and d) epidemic, but the number of people requiring hospital care is considerably fewer at any time. Patients in the $I_{{{\text{ps}}}}$ stage infect on average 4.5 individuals; hospital capacity is $Y_{3} = 70,000$. $t_{0} = 5$, $t_{2} = 8$ (days) The initial values for the dynamics are $S\left( 0 \right) = 10^{7}$, $E\left( 0 \right) = 100$, $I_{{{\text{ps}}}} \left( 0 \right) = I_{{\text{s}}} \left( 0 \right) = I_{{\text{h}}} \left( 0 \right) = R\left( 0 \right) = 0$. All other parameters of the dynamics are listed in Table 1 and in Supplementary Table 2 and 3.

Transmission rate depends on the local setting (venue) (Fig. 1 and see details in the “Method” section), consequently we calculated overall transmission rate and reproduction number based on venue specific transmission probabilities dependent on the density of people present, the location specific secondary attack rate and the mean time people spend in such setting. By closing certain (controlled) portion of such venues, the transmission rate can be considerably reduced (Fig. 3), and thus the spread of the epidemic managed.

**Figure 4:**
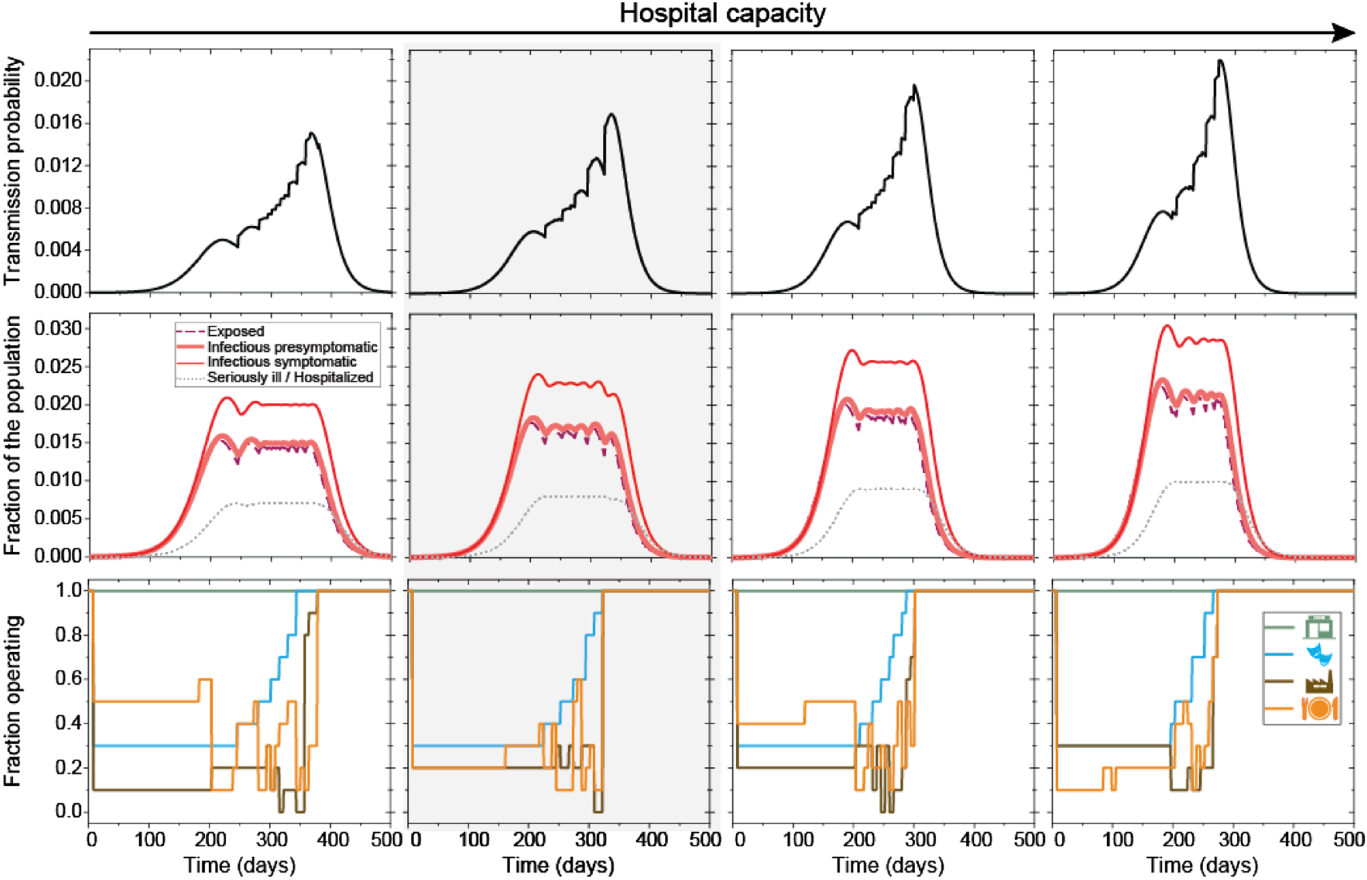
The effect of the number of available hospital beds on the course of the controlled epidemic. Transmission probability (first row) can be controlled by the selective (partial) closing or opening of certain venues (economic sectors) (last row). With more available hospital beds (columns, increasing from left to right), the epidemic lasts for a shorter time (middle row). Patients in the ${I}_{\mathrm{ps}}$ stage infect on average 4.5 individuals; hospital capacity is ${Y}_{3}=70,000;80,000;90,000$ and $100,000$ in the columns, respectively. Results with shaded backgrounds correspond to the solution of the game, which is also depicted in Fig. 3. Parameters are otherwise as in Fig. 3.

**Figure 5:**
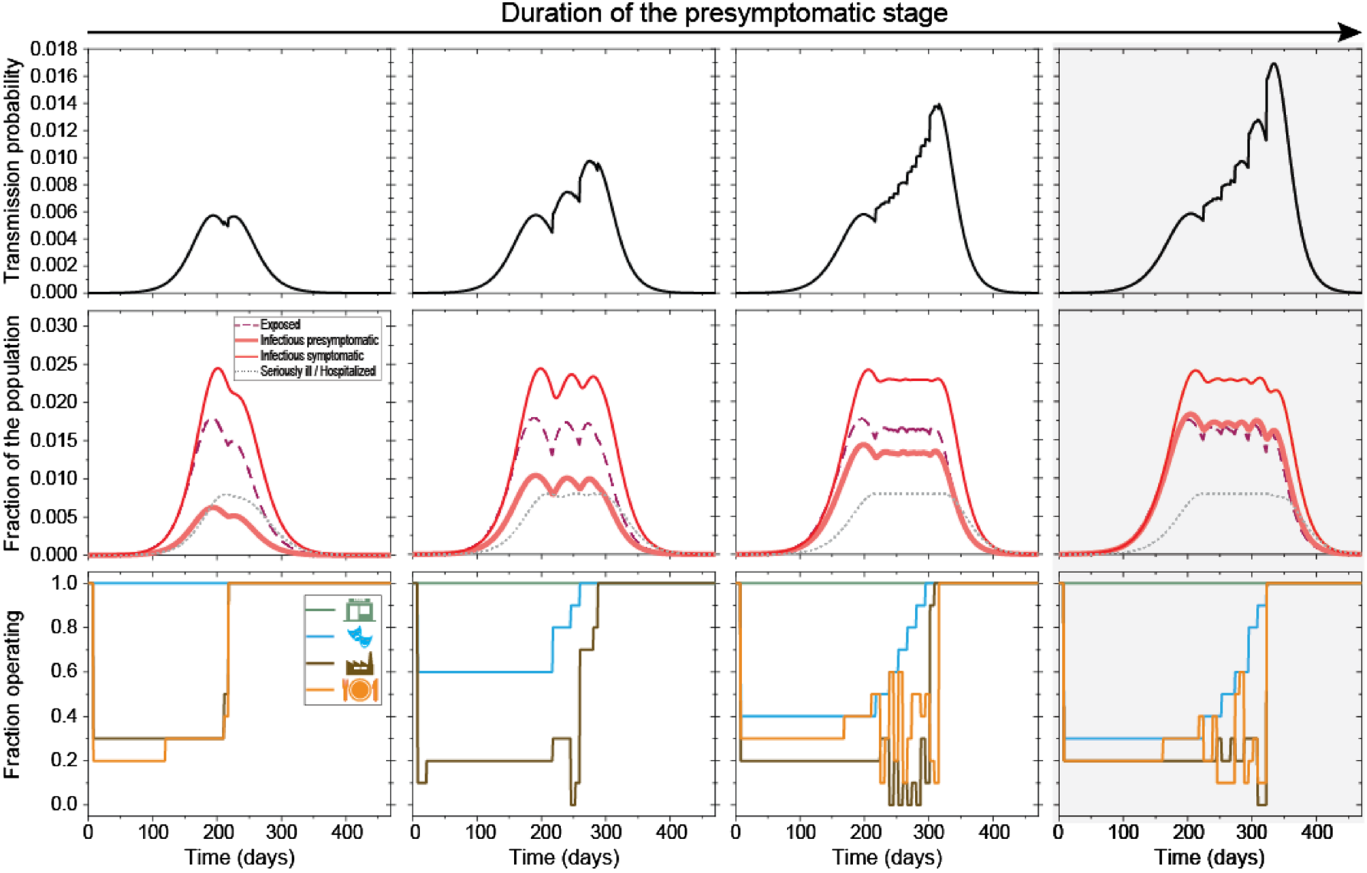
The effect of the duration of the presymptomatic stage on the course of the controlled epidemic. Transmission probability (first row) can be controlled by the selective (partial) closing or opening of certain venues (economic sectors) (last row). With a longer presymptomatic stage (columns, increasing from left to right), the epidemic lasts for a shorter time (middle row), and a higher portion of the economic activities need to be restricted (last row) to avoid the overflowing of the hospital capacities. Patients in the ${I}_{\mathrm{ps}}$ stage infect on average 1.5; 2.5; 3.5 and 4.5 individuals corresponding to ${t}_{1}=1.393;2.3217;3.2504$ and $4.1791$ days in the columns, respectively (see Supplementary Table 3). Results with shaded backgrounds correspond to the solution of the game, which is also depicted in Fig. 3. Parameters are otherwise as in Fig. 3.

With weekly management of the portion of the four illustrative economic sectors under (partial) lockdown (Fig. 3f), the state can keep the number of patients requiring hospitalization under its maximal capacity (Fig. 3d), thus fulfilling its political goal. The optimal control involves a different mix and degrees of closures, indicating that “one size fits all” type of interventions is not optimal, they have to be tailored to the situation. Please note, for example, that stores are not required to close, thus the political goal of having key stores open is automatically fulfilled. Moreover, while we have included a boundary condition requiring a large enough workforce to be available, the number of sick people was never risking continuous operation of the key facilities when the spread of the disease was controlled. On the other hand, without control, the epidemic would run its course faster, but the hospital capacity would be quickly overwhelmed (compare Fig. 3c with d). Increasing hospital capacity allow for shorter controlled period (the period after which lockdowns are lifted entirely) (Supplementary Table 5 and Fig. 4). Longer presymptomatic stage of the infected translates to higher effective reproduction number (*R*_E_), which requires longer closures of controlled venues (Fig. 5).

The state control decreases economic production. Fewer hospital beds necessitate stricter control, which may have serious economic consequences. With the end of the non-pharmaceutical interventions affecting whole sectors, the epidemic is far from over as there are still infected individuals in quarantine and there are still patients in the hospitals. It could take months before hospitalization rates drop to significantly low levels (Supplementary Table 6). Higher number of available hospital beds and a shorter presymptomatic stage shorten the length of the epidemic (Figs. 4 and 5).

The total number of hospital days increases with the length of the presymptomatic period. Contrary to the monotone effects so far, the number of hospital days does not always change monotonically with increasing hospital bed capacity (Supplementary Table 7). Consequently, the final cost to the state, calculated with the loss of Gross Value Added due to lockdowns, the cost of increasing hospital bed capacity and the increased burden on the health care system, is not monotonic with hospital bed capacity. An intermediate number of hospital beds minimizes costs.

**Figure 6:**
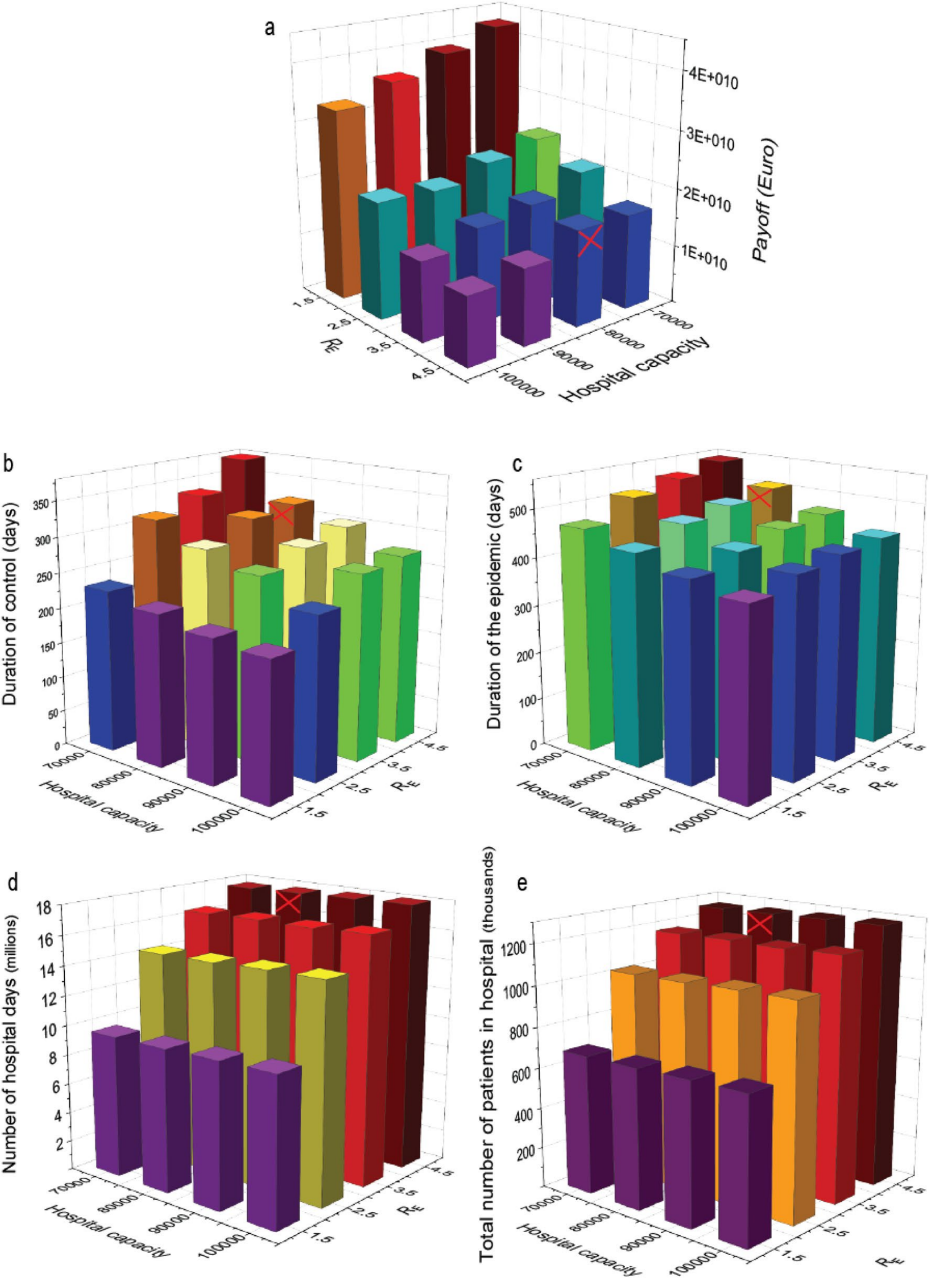
Characteristics of the economic effects of epidemic and the payoff as function of the strategies. (**a**) The payoff of the state (Supplementary Table 9). Colors are visual aids to assess height of the bars. The optimal solution is at 800,000 beds for the worst case of a pathogen having ${R}_{\mathrm{E}}=4.5$. The bar corresponding to this case is marked with red crossed lines. In the calculations, the payoff depended on the following “background variables”: (**b**) Duration of the control (Supplementary Table 5); (**c**) duration of the whole epidemic (Supplementary Table 6); (**d**) total number of hospital days during the epidemic (Supplementary Table 7); (**e**) total number of patients in hospital during the epidemic (Supplementary Table 8). Clearly, these background variables depend on the characteristic of the pathogen and the available hospital capacity, and thus affect the economic effect of the epidemic.

The state should be prepared to face an unknown pathogen. While its characteristics are mostly unknown, the pathogen is such that it would cause a pandemic; and at the same time the disease it causes is not too fatal so that hospitalization helps patient to recover. Furthermore, while the state cannot foretell the exact characteristic of a novel pathogen, it can have some educated guesses based on other known pathogens (see section 1.1 in the SI). In such case, the state should adopt Wald’s paradigm of *maximin* or *pessimistic solution* (also called conservative solution)^45^: it counts with the “worst case”, supposing a strategy choice of the pathogen that minimizes the payoff (revenues) of the state, and the latter maximizes this minimum. In other words, prepare for the most damaging strategy choice of the pathogen that can be evolved, and choose the optimal hospital capacity accordingly. The revenues of the state are minimized with a more severe disease (here longer duration of the pre-symptomatic stage), but then among these minimum outcomes, the maximum is obtained for an intermediate number of beds. Our numerical investigation thus reveals that, in general, not the maximum hospital capacity is the optimal solution for the game conflict (Fig. 6). The state should not try to increase hospital bed capacity recklessly, as its cost could be minimized with fewer beds and still being able to provide hospitalization to all needing citizens.

As transmission rates depends on the venues and the costs (decrease in revenues) depend on the economic structure of a given country, even if two countries have the same political objective, namely provide hospital care to all needing patients and keeping the extent of the lockdown as low as possible, they may have different hospital extension strategies, according to their different economic structures. But our results have proved to be robust to changes in the revenues and costs: a tenfold increase or decrease in the weight of any one sector and a fourfold increase or decrease in the cost associated with health care does not change the solution of the game against Nature.

## Discussion

We introduced a game-theoretical framework to analyse how a state can prepare itself to the emergence of a new pathogen. With an extended SEIR epidemiological model incorporating location specific transmission probabilities we calculated optimal control of the pathogen transmission for various combinations of pathogen characteristic and state preparedness (number of hospital beds). We showed that even if the state prepares for the worst case, it should not opt for the largest possible extension of hospital capacities.

Our model is unique in combining game theory and optimal control in an epidemiological study. It is obvious that when a new pathogen emerges, there is no corresponding vaccination available, therefore the state is not able to control the pandemic by allocating vaccination or antidotes^46,47^. Previously, optimal control of pathogen transmission has been investigated with a flat transmission rate^12,48–50^. In our model, different locations have different, site-specific transmission rates. As local circumstances critically affect secondary attack rates^51^, the transmission model is mechanism based since the study of the transmission rate is not based on a given dynamics^52,53^. With this finer structure, economic sectors can be controlled differently in a meaningful way from an epidemiological point of view. There could be various political goals for the optimal control, such as the combined minimization of the weighted combination of death due to the pathogen, and the strength or cost of the control of the transmission coefficient^49,54^; or the minimization of the maximum fraction infected during the epidemic^48^. In comparison, our more complex modelling approach takes the optimal control (minimization) of the restriction as a first step, describing a possible public health policy of the state. Our evolutionary game model is built upon the solution of this optimal control model, and the strategy of the state in the game against the pathogen is hospital capacity.

Game theory has been employed in epidemiology^55^, these models deal with individual choices, for example whether or not to follow an intervention policy (like vaccination), and the studies estimate the effects of these individual decisions on the overall epidemic spread^46,55^. Unlike these studies, our investigation is aimed at the introduction of a game played between the pathogen and the state, where the state can efficiently enforce restrictions, thus we did not consider individual differences in rule-abiding behaviours. In our model, the state is a player^55–59^. A large number of payoff functions have already been studied, where the payoff usually is the combination of two objectives: Minimize the number of infections (and hence the number of deaths), and minimize the economic loss^58,60–65^. A state, however, may have purely socio-political objectives besides or instead of the above ones. The requirement of minimizing social restriction, but at the same time guaranteeing hospital care to all needed patients in our model is such socio-political objective.

The state can increase hospital capacity, for example, by using mothballed military stockpiles. The hospital capacity coupled with controlled transmission affects both the duration of the epidemic and the number of infected persons, also influencing in this way the economic effect of the epidemic^66,67^. The other participant in the game is a new pathogen, which is not a rational player. (From evolutionary aspect, the objective of the pathogen is its long-term survival, realized by mutations. This may be subject of further study.) The pathogen is not necessarily looking to minimize the objective of the government. In other words, there is no biological reason to consider a zero-sum game. In our setting, the government is a distinguished player, finding the best policy when the worst scenario is realized. The properties (strategies) of the emerging pathogen have been chosen on the basis of the parameters of known pathogens. As a matter of fact, our game model is a particular one, a “game against Nature”. In such games the behaviour (strategy choice) of the pathogen (Player 1, symbolically called “Nature”) cannot be influenced, the “solution” is considered only from the viewpoint of Player 2. To our knowledge, our model is the first in the game-theoretical modelling of epidemic processes where the socio-political objectives of the state are taken into account.

Time is a key parameter in dealing with an epidemic. The time constraints of the epidemiological dynamics, such as the mean duration of the presymptomatic stage, directly affect the transmission rate of the pathogen. Time also has an indirect role, namely the choice of hospital capacity, through the control of the epidemic influences the duration of the economic restrictions, the number of infected persons and the duration of the epidemic as well.

A realistic long-term scenario would be a sequence of mutations of the pathogen, with subsequent strategy choices of the state, which could be modelled with a dynamic Stackelberg leader–follower game^68^, where the leader is the evolution, producing a new pathogen and then the state as follower moves sequentially. Nevertheless, as a first step in developing this framework, we analysed the corresponding one-shot game (“game against Nature”). We presented a multidisciplinary game-theoretical models that can simultaneously handle the knowledge on evolutionary epidemiology (concerning the possible properties of a recently evolved pathogen), the political objectives of a state and the economic effect of the non-pharmaceutical interventions against the epidemic to reach these goals. Since COVID-19 demonstrated that epidemiological models can play an important role in the planning of anti-epidemic measures of a state^16^, models like ours can help the preparation to the defence against future epidemics, namely by in silico simulation of the economic effect of an anti-epidemic defence against a new mutant pathogen.

## Supplementary Information

Supplementary Information 1.

Supplementary Information 2.

## Data Availability

The optimal control data generated and analysed in this study are included in this published article (and its Supplementary Information files).
