# Supplementary material for: State-controlled epidemic in a game against a novel pathogen
Source: Sci Rep. 2022 Sep 20;12:15716. doi: 10.1038/s41598-022-19691-7 (PMC9488893; doi:10.1038/s41598-022-19691-7)
Supplement: Supplementary file 1 — Supplementary Information 1. [file 41598_2022_19691_MOESM1_ESM.docx]

**State-controlled epidemic in a game against a novel pathogen**

József Garay, Ádám Kun, Zoltán Varga, Manuel Gámez, Ana Belén Castaño-Fernández, Tamás F. Móri

**Supplementary Information**

The supplementary information supplements the method description of the main text. Here we first elaborate on the game between the novel pathogen and the state (section 1); then present the derivation of the change of a pandemic overspilling to other countries (section 2); and finally derive the venue-dependent transmission probability (section 3).

# 1. Details of the game of the state against a pathogen

Here we give an exemplar parametrization of the model presented so far. The characteristic of the pathogen is based on real-life pathogens detailed in Section SI.1.1. Then we chose four board categories of venues or sections of the economy that were often closed or limited during the COVID-19 pandemic by the state, to illustrate the control the state can exert to manage the epidemic (SI.1.2.). Then we show results assuming different costs (SI.1.3.). We have not tried to model any particular pathogen or country; we just give examples of how a game theoretical framework can help states deal with an emergent disease.

## 1.1. The pathogen

The novel pathogen causing an epidemic that the state wants to control has epidemiological parameters which are similar to known pathogens that had caused epidemics. Here we collect data on influenza, the first SARS pandemic, and COVID-19 (Supplementary Table 1) as all three is known to cause a pandemic. The following data is not meant to be a systematic review of the literature, nor a meta-analysis, we only wanted to approximate real pathogens. The epidemiological model is parametrized according to the “novel pathogen” row of Supplement­ary Table 1. Where a range is given, we have explored different parameter combinations.

The novel pathogen is assumed to have a non-symptomatic infectious phase like influenza and COVID-19. We explored a wider range of parameters here, as preliminary analysis showed that this phase is key in spreading the disease to other areas as well as a hindrance to mitigation efforts. Time to recovery is set to values around what we observed for COVID-19 and SARS, the longer hospitalization times create a strain on the health care system. We further assume that the disease caused by the novel pathogen is such that existing medical practice can increase the recovery rate of hospitalized, severely, or critically ill patients. Such intervention might not be available in case of a new, unknown disease. We explored various death rates: recovery from the hospitalized stage is varied between 10% and 90%, meaning case fatality rates are between 1.4% and 12.6%.

Supplementary Table 1. Epidemiological parameters of selected pathogens/diseases

|  | *t*_0_ | *t*_1_ | *θ*_1_ | *t*_2_ | *θ*_2_ | *t*_3_ | *θ*_3_ | *R*_0_ |
| --- | --- | --- | --- | --- | --- | --- | --- | --- |
| Influenza | 1 | 2 | 0.88 | 4 | 0.87 | 6 | 0.9 | 1.3–25 |
| COVID-19 | 3 | 2 | 0.3 | 8 | 0.8 | 14 | 0.9 | 2–3.5 |
| SARS | 4.6 | 0 | 0 | 10 | 0.5 – 0.6 | 14 – 16 | 0.19 | 2.2 – 2.6 |
| **Novel pathogen** | 5 | 1 – 4.2 | 0.3 | 8 | 0.8 | 14 | 0.1–0.9 | 1–5 |

### Influenza

Influenza is a seasonal disease caused by different strains of the influenza virus. The incubation period is reported to be 3 days, and the mean time to peak infectiousness is two days, which means that considerable infection can happen pre-symptomatically[^1^](#_ENREF_1). Symptoms might not develop at all, only 46% of the serologically confirmed influenza cases have influenza-like symptoms, including fever[^2^](#_ENREF_2). And a meta-analysis found that the prevalence of asymptomatic carriage (absence of symptoms) is between 5.2–35.5%; and the prevalence of subclinical cases ranges from 25.4–61.8% [^3^](#_ENREF_3). Consequently, we can assume 1 day of incubation period, 2 days of asymptomatic infectious stage, and 4 days of symptomatic infectious phase[^4^](#_ENREF_4). 50% of the infected develop severe symptoms (severe enough not to go to work/school), this might be up to 75% in the elderly[^4^](#_ENREF_4), but the hospitalization rate is only 1.5% of the infected[^5^](#_ENREF_5). The death rate is estimated to be 0.13%, and 9.1% of hospitalized patients die[^5^](#_ENREF_5).

### COVID-19 / SARS-CoV-2

The characteristics of the COVID-19 pandemic caused by SARS-CoV-2 are beginning to be known. There is variation in the parameters which is probably caused by the differences in demography, population density, and the capabilities of the healthcare system.

The incubation period was found to be 6.1 days (interval 1–16)[^6^](#_ENREF_6); 6,4 days (interval 2–11 days)[^7^](#_ENREF_7); 5,2 days[^8^](#_ENREF_8); 5,0 days[^9^](#_ENREF_9); 4,0 days[^10^](#_ENREF_10); and 5,1 days[^11^](#_ENREF_11). Individuals are probably not infectious for most of this time [^12^](#_ENREF_12), but at the end of this period, individuals could be infectious.

Asymptomatic infection is possible[^13-17^](#_ENREF_13), and their viral load can be as high as that of symptomatic individuals[^18^](#_ENREF_18). Some of these are presymptomatic, i.e. will develop symptoms later. One US case study found that 13% (3/23) of the SARS-CoV-2 infected individuals do not develop any symptoms. A study of 24 asymptomatic individuals (identified by contact tracing) in Nanjing, China, resulted in 7 individuals not developing any symptoms later, nor having abnormal CT images (29% asymptomatic)[^19^](#_ENREF_19). These individuals were virus positive for a median of 4 days[^19^](#_ENREF_19). 29% (16/55) of initially asymptomatic individuals remained so during hospitalization in Shenzhen, China[^20^](#_ENREF_20). 8% (4/49) of initially asymptomatic individuals remained so during hospitalization in Guangzhou, China[^21^](#_ENREF_21). There is a report of 5% (13/262) asymptomatic from Beijing[^22^](#_ENREF_22). 50–75% of the infected were asymptomatic in the Italian village of Vō^[23](#_ENREF_23)^. For the Diamond Princess cruise ship it was estimated that 17.9% of the infected were asymptomatic[^24^](#_ENREF_24). 30% asymptomatic infectious individuals were also estimated for the Japanese citizens evacuated from Wuhan [^25^](#_ENREF_25). In a call centre in South Korea, all infected were followed and 4.1% (4/97) remained asymptomatic[^26^](#_ENREF_26). A systematic review of the rate of asymptomatic SARS-CoV-2 infection found it to be 17% [^27^](#_ENREF_27). Zhou *et al.* [^28^](#_ENREF_28) warn that the truly asymptomatic individuals (1/328 in Shanghai) might have a subclinical infection, and might not be able to infect others (a similar outcome was reported in [^26^](#_ENREF_26)), albeit cluster analysis of infection pathways indicates otherwise[^29-31^](#_ENREF_29). Consequently, we assume 3 days of non-infectious incubation period and 2 days of pre- or asymptomatic infectious period. Here we assume 30% asymptomatic, i.e. individuals, who recover after the infectious non-symptomatic phase. As presented above, there is a huge variation in the estimate of this key parameter.

Patients die 14 or 22 days after symptom onset, thus it has a bimodal distribution[^32^](#_ENREF_32). Italian data show that four days pass between symptom onset and hospitalization, and in another five days patients may die (<https://www.epicentro.iss.it/coronavirus/bollettino/Report-COVID-2019_24_marzo_eng.pdf>). Other Chinese data indicate 22 days (IQR 18–25 days) from illness onset to discharge from the hospital and 18.5 days (IQR 15–22 days) to death[^33^](#_ENREF_33). Invasive mechanical ventilation started at day 14.5 (IQR 12–19) from the onset of symptoms. In another case study, a median of 7 days (IQR 4–8) passed between symptoms onset and hospitalization, and another 3 days to admission to the intensive care unit. In yet another study, hospital admission time was 3–4 or 5–9 days, and patients died after 15 from illness onset[^9^](#_ENREF_9). The mean duration of hospitalization was 12 days[^10^](#_ENREF_10). The median time from the first symptom to dyspnea (shortness of breath) was 5 days, another 7 days passed to hospital admission, and yet another 8 to intensive care admittance[^34^](#_ENREF_34). According to the WHO-China joint mission’s report[^35^](#_ENREF_35) collecting data from China till the end of February, 80% of the identified infected individuals have mild disease, 14% of the infected develop severe and 6% of them critical conditions. The severe and critical condition develops 7 days after symptoms onset. Recovery can last for 2 weeks in mild cases, and 3–6 weeks in severe cases. In Europe (according to the ECDC[^36^](#_ENREF_36)), 32% of the identified cases required hospitalization, and 2.4% required respiratory support or ventilation. The crude fatality rate was 1.5% among identified cases, and 11% among hospitalized. In summary, we set 8 days as the mean time to recovery from a mild infection, and 80% of the symptomatic individuals recover this way. The duration of hospitalization is on average 14 days, and 90% of the patients recover.

*R*_0_ is estimated to be 2.2 [^8^](#_ENREF_8)^,^[^37^](#_ENREF_37); 2.56 [^38^](#_ENREF_38); 2.5 [^39^](#_ENREF_39); 2.24–3.58 [^40^](#_ENREF_40); 2.28 [^41^](#_ENREF_41); and 2.8–3.3 [^42^](#_ENREF_42), 2.76 – 3.25 [^36^](#_ENREF_36).

Our model assumes that recovered individuals acquire immunity to the infection at least to the degree that they won’t be considered susceptible again within the time frame of the current epidemic. There was a fear that immunity against SARS-CoV-2 only lasts for a very limited time[^43^](#_ENREF_43), but a robust immune response can be found several months after the first infection [^44^](#_ENREF_44).

### SARS

The SARS pandemic of 2002/2003 [^45-47^](#_ENREF_45) originated in Guangdong, China, and subsequently spread to 32 countries worldwide. There were 8437 identified cases of which 813 died. The death rate is thus 9.6%. As all infected individuals were isolated in a hospital, thus admission times mostly reflect the time a case was found.

The incubation period was estimated to be 6.37 days[^48^](#_ENREF_48) during the epidemic, which was revised to 4.6 days[^49^](#_ENREF_49) when all known cases in Hong Kong were considered.

Contrary to influenza and COVID-19, infectiousness only started after the onset of symptoms[^50^](#_ENREF_50). This was probably a key factor in the containment of the pandemic. Peak infectiousness was observed 7 to 8 days following the onset of clinical symptoms[^45^](#_ENREF_45), but an infectious period of only 5–6 days is also reported[^51^](#_ENREF_51).

50% of the infected developed ARDS and required intensive care[^52^](#_ENREF_52); 32% required intensive care (20% mechanical ventilation)[^53^](#_ENREF_53); 61% of the patient required respiratory assistance, and 11% required mechanical ventilation[^54^](#_ENREF_54). We thus assume that 50–60% of the patient can recover without hospitalization (please note that infectious people were mostly hospitalized to contain the epidemic), and the mean time in this phase is then between 2–8 days[^49^](#_ENREF_49) or 10 days[^54^](#_ENREF_54).

The duration of hospitalization, i.e. from admission to discharge (recovery) is reported to be 23.5 days[^48^](#_ENREF_48) and 26.5 days[^49^](#_ENREF_49). The mean time from onset to death was 23.7 days[^49^](#_ENREF_49), albeit the mean 35.9 days from hospital admission to death was also reported[^48^](#_ENREF_48). We calculated that the first 10 days is just the infectious symptomatic period cited above (that people spent quarantined in the hospital), and the rest of the 24–26 days is the required hospitalization. To arrive at the observed 9,6% case fatality rate, only 19.2% of the hospitalized patient recover.

## 1.2. Illustrative venue types and economic consequences

The four board venue types represent ones having different transmission probabilities and different contributions to the economy. We assume that individuals spend most of their time either at work or at home. Otherwise, they shop, participate in entertainment or go visit places. These times (Supplementary Table 2) represent assumed averages.

Supplementary Table 2. Parameters of pathogen transmission for different venue types

| Venue | $n_{i}$ | $k_{i}$ | $\alpha_{i}$ | $\tau_{i}$ |
| --- | --- | --- | --- | --- |
| Retail stores | 20 | 2 | $5\alpha$ | 1$\times$4 |
| Arts, entertainment and recreation | 100 | 10 | $10\alpha$ | 2$\times$4 |
| Manufacturing | 50 | 5 | $\alpha$ | 10$\times$4 |
| Travel, restaurants, hotels | 200 | 10 | $\alpha$ | 1$\times$4 |
| Home | * | * | * | 10$\times$4 |

Given the above parameters, the number of susceptible infected by an infectious presymptomatic individual is given in Supplementary Table 3. The herd immunity threshold is also given based on the calculated basic reproductive number.

Supplementary Table 3. Duration of the presymptomatic infectious stage and herd immunity threshold for different parameters of the dynamics of the epidemic

| $R_{E}$ | $\alpha$ | $t_{1}$ | Herd immunity threshold (*K_i_*) |
| --- | --- | --- | --- |
| 1.5 | 0.001 | 1.3930 | $1/3$ |
| 2.5 | 0.001 | 2.3217 | $3/5$ |
| 3.5 | 0.001 | 3.2504 | $5/7$ |
| 4.5 | 0.001 | 4.1791 | $7/9$ |

The cost of these measures is the loss of gross value added. Gross value added (GVA) is – briefly – GDP without the effect of taxes and redistributions/subsidies, this is a better measure of the effect of a sector than GDP.

Supplementary Table 4. Total returns by sectors, for the model population of 10^7^ inhabitants

| Venue | Gross Value per capita (Euro) | Total Returns, $g_{i}$  (yearly in Euro) |
| --- | --- | --- |
| Retail stores excluding ones selling food, toiletries and drugs | 572 | 572×10^7^ |
| Arts, entertainment and recreation | 921 | 921×10^7^ |
| Manufacturing | 3470 | 3470×10^7^ |
| Tourism | 1940 | 1940×10^7^ |

We used the average per capita GVA of the European Union (28 member countries) for the above sectors extracted from Eurostat (European Statistical Office) (<https://ec.europa.eu/eurostat>). The value added at factor cost for different aggregates of activities, classified by NACE Rev. 2 was extracted as detailed below. We used data published for 2017, as was the most recent year in which all data were available at the time of writing (June 2020).

**Arts, entertainment and recreation venues** are represented by gross value added of Section R-U. Only aggregate data for these sections are available, albeit it contains parts that should be excluded. Section R is Arts, Entertainment and Recreation; Section S is other service activities; Section T is Activities of households as employers; Undifferentiated goods- and services-producing activities of households for own use; and Section U is Activities of extra-territorial organizations and bodies. If detailed data were available then most of Section R would be listed here, and some of section S, like hairdressers, small repair shops, etc. would be listed with retail stores.

**Tourism** is represented by the sum of the following activities:

- Section H, Group 50.1 Sea and coastal passenger water transport
- Section H, Group 50.3 Inland passenger water transport
- Section H, Group 51.1 Passenger air transport
- Section H, Class 52.22 Service activities incidental to water transportation
- Section H, Class 52.23 Service activities incidental to air transportation
- Section I Accommodation and food service activities
- Section N, Group 79 Travel agency, tour operator and other reservation service and related activities

These economic activities include passenger travel that predominantly used by tourists, services of restaurants, hotels and travel agencies. There are places where water or air transport is part of everyday commuting, and accommodation could also cater for workers and other business-related activities, furthermore restaurants might sell take-away food, but the grain of the economic data does not allow us to make such detailed distinction.

**Manufacturing** is represented by Section C - Manufacturing, except for

- Section H, Division 10 Manufacture of food products
- Section H, Division 11 Manufacture of beverages
- Section H, Division 21 Manufacture of basic pharmaceutical products and pharmaceutical preparations
- Section H, Division 33 Repair and installation of machinery and equipment

Here we assumed that industries producing food and drinks, as well as medicine and other medical products are deemed essential and cannot be shut down, and repair and maintenance in other industries (e.g. agriculture) also must function. Other factories can be closed if necessary.

**Retail stores** are represented by Section G Division 47 „Retail trade, except of motor vehicles and motorcycles”. As retail stores and markets selling food and drinks, and pharmacies cannot be closed down (represented by the maximum control on stores), we have excluded the following from the calculation:

- Section G Group Class 47.11 Retail sale in non-specialized stores with food, beverages or tobacco predominating
- Section G Group Group 47.2 Retail sale of food, beverages and tobacco in specialized stores
- Section G Group Class 47.21 Retail sale of fruit and vegetables in specialized stores
- Section G Group Class 47.22 Retail sale of meat and meat products in specialized stores
- Section G Group Class 47.23 Retail sale of fish, crustaceans and molluscs in specialized stores
- Section G Group Class 47.24 Retail sale of bread, cakes, flour confectionery and sugar confectionery in specialized
- Section G Group Class 47.25 Retail sale of beverages in specialized stores
- Section G Group Class 47.29 Other retail sale of food in specialized stores
- Section G Group Class 47.73 Dispensing chemist in specialized stores
- Section G Group Class 47.74 Retail sale of medical and orthopaedic goods in specialized stores
- Section G Group Class 47.75 Retail sale of cosmetic and toilet articles in specialized stores
- Section G Group Class 47.81 Retail sale via stalls and markets of food, beverages and tobacco products

**Cost of hospitalization.** We assume that the cost of a new hospital bed (including all other equipment needed) is $h_{0}=$ 10 000 €/bed. The daily cost of hospitalization is $h_{1}=$ 1 500 €/day.

## 1.3. Technical details of the game of the state against a pathogen

The strategy of the pathogen is $t_{1}$ the mean time of the presymptomatic infectious state and the strategy of the state is the level of extra hospital capacity it installs ($Y_{3}$). The payoff to Player 2 is defined as the difference between the total income and total costs, for the whole duration of the epidemic, corresponding to the strategy pair $(t_{1},Y_{3})$:

$$f\left( t_{1},Y_{3} \right)=\sum_{i=1}^{4} \frac{g_{i}}{365}\int_{0}^{T_{1}} u_{i}^{*}\left( t \right)dt+\left( T_{3}-T_{1} \right)\sum_{i=1}^{4} \frac{g_{i}}{365}-h_{0}\left( Y_{3}-Y_{0} \right){-h}_{1}\int_{0}^{T_{3}} I_{h}\left( t \right)dt. \text{(SI.1)}$$

Here $g_{i}$ is the gross value added for venue (sector) *i*. The first term thus measures the income of the state modified by the control, i.e. if there are extensive lockdowns and restrictions then there is less income from those economic activities. To calculate the payoff, for every fixed pair $\left( t_{1},Y_{3} \right)$, we need the optimal controls obtained from the numerical investigation (see examples in Figure 4f, and in Figures 4 and 5).

From the economic point of view, the epidemic lasts for the duration *T*_1_*_,_* (Supplementary Table 5) after which all controls $u_{i}$ equal 1, i.e. there are no restrictions. On the other hand, from a public health perspective, the epidemic ends at time *T*_3_ (Supplementary Table 6), when the number of hospitalized patients decreases below $Y_{3}/1000.$ During this time ($T_{3}-T_{1}$) the economy is fully functional, but there could still be extra costs as patients are hospitalized. This is the second term in the equation. The first two terms are the income for the state.

The third term is the cost of new hospital beds ($Y_{3}-Y_{0}$), and the fourth term is the cost of hospitalizations. $I_{h}(t)$ is the number of patients in hospital at time *t*; and $C=\int_{0}^{T_{3}} I_{h}(t)dt$ is the total number of hospital days during time $T_{3}$ (Supplementary Table 7).

Supplementary Table 5. Time duration *T*_1_ (days) of effective optimal control, for different pairs$\left( \boldsymbol{t}_{\boldsymbol{1}}\boldsymbol{,}\boldsymbol{Y}_{\boldsymbol{3}} \right)$

| $Y_{3}$  $t_{1}$ | 70 000 | 80 000 | 90 000 | 100 000 |
| --- | --- | --- | --- | --- |
| 1.393 | 231 | 217 | 203 | 196 |
| 2.3217 | 315 | 287 | 266 | 231 |
| 3.2504 | 336 | 315 | 287 | 266 |
| 4.1791 | 378 | 322 | 301 | 273 |

**Supplementary Table 6**. **Time duration *T*_3_ (days) of the epidemic, for different pairs** $\left( \boldsymbol{t}_{\boldsymbol{1}}\boldsymbol{,}\boldsymbol{Y}_{\boldsymbol{3}} \right)$ **under optimal control**

| $Y_{3}$  $t_{1}$ | 70 000 | 80 000 | 90 000 | 100 000 |
| --- | --- | --- | --- | --- |
| 1.393 | 468 | 441 | 413 | 392 |
| 2.3217 | 511 | 475 | 440 | 418 |
| 3.2504 | 532 | 492 | 461 | 431 |
| 4.1791 | 553 | 509 | 470 | 440 |

Supplementary Table 7. Total number of hospital days, *C*, for different pairs $\left( \boldsymbol{t}_{\boldsymbol{1}}\boldsymbol{,}\boldsymbol{Y}_{\boldsymbol{3}} \right)$

| $Y_{3}$  $t_{1}$ | 70 000 | 80 000 | 90 000 | 100 000 |
| --- | --- | --- | --- | --- |
| 1.393 | 9,576,238 | 9,644,540 | 9,781,398 | 9,888,441 |
| 2.3217 | 14,307,948 | 14,393,803 | 14,572,379 | 14,660,934 |
| 3.2504 | 16,446,920 | 16,547,311 | 16,546,698 | 16,707,823 |
| 4.1791 | 17,672,581 | 17,772,485 | 17,881,997 | 17,979,286 |

Since the average duration for a patient in a hospital is $t_{3}=14$, the total number of patients in hospitals during time $T_{3}$ is C/14, as shown in Supplementary Table 8.

Supplementary Table 8. Total number of patients in hospitals during time $\boldsymbol{T}_{\boldsymbol{3}}$ for different pairs $\left( \boldsymbol{t}_{\boldsymbol{1}}\boldsymbol{,}\boldsymbol{Y}_{\boldsymbol{3}} \right)$

| $Y_{3}$  $t_{1}$ | 70 000 | 80 000 | 90 000 | 100 000 |
| --- | --- | --- | --- | --- |
| 1.3930 | 684,017 | 688,895 | 698,671 | 706,317 |
| 2.3217 | 1,021,996 | 1,028,128 | 1,040,884 | 1,047,209 |
| 3.2504 | 1,174,780 | 1,181,950 | 1,181,907 | 1,193,415 |
| 4.1791 | 1,262,327 | 1,269,463 | 1,277,285 | 1,284,234 |

The payoff values $f\left( t_{1},Y_{3} \right)$, corresponding to strategy pairs $\left( t_{1},Y_{3} \right)$, calculated according to Equation (SI.1), are shown in Supplementary Table 9.

Table 9. Payoff values $\boldsymbol{f}\left( \boldsymbol{t}_{\boldsymbol{1}}\mathbf{,}\boldsymbol{Y}_{\boldsymbol{3}} \right)$

| *Y*_3_  $t_{1}$ | 70 000 | 80 000 | 90 000 | 100 000 |
| --- | --- | --- | --- | --- |
| 1.393 | 42,680,927,328 | 39,433,417,767 | 36,079,433,650 | 33,054,683,662 |
| 2.3217 | 24,395,701,472 | 22,374,798,494 | 19,726,785,980 | 20,200,965,523 |
| 3.2504 | 21,094,560,494 | 17,791,046,502 | 16,452,209,593 | 13,722,515,766 |
| 4.1791 | *16,459,598,860* | ***16,611,177,105*** | *13,126,953,087* | *11,794,911,941* |

In the context of this “game against nature”, for Player 2 it is at hand to adopt Wald’s paradigm of *maximin* or *pessimistic* *solution* (also called conservative solution), see e.g. [^55^](#_ENREF_55): Player 2 counts with the “worst case”, supposing a strategy choice of Player 1 that minimizes the payoff of Player 2, and the latter maximizes this minimum. Considering the values $f\left( t_{1},Y_{3} \right)$ as entries of a corresponding 4×4 payoff matrix $M=[m_{jk}],$ formally, $k_{0}$ is a *maximin solution for Player 2*, if

$$\max_{k} \min_{j} m_{jk}=\min_{j} m_{jk_{0}}.$$

In our present case, for $k=1, 2, 3, 4$, in the $k$*-*th column of Supplementary Table 9 $\min_{j} m_{jk}$ is highlighted, and $\max_{k} \min_{j} m_{jk}$ is obviously reached at $k_{0}=2$. Therefore the $Y_{3}=80 000$ is the maximin or pessimistic strategy for Player 2. (In terms of indices, the corresponding strategy pair is (4,2).)

We note that the maximin solution for Player 2, $k_{0}=2$, is not an extremal strategy (either $Y_{3}=70 000$, or $Y_{3}=100 000$ beds). Intuitively, the reason for it might be trade-off between available beds shortening the epidemic (and thus the economic cost associated with the lockdowns), but at the same time increasing the total cost of health care.

Table 10. Maximin solutions for proportionally changed economic parameters

| $g_{i}$  $h_{i}$ | $10g_{i}$ $(i=1,2,3,4)$ | $\frac{g_{i}}{10}$ $(i=1,2,3,4)$ |
| --- | --- | --- |
| $4h_{i}$ $(i=1,2)$ | (4,2) | (4,2) |
| $\frac{h_{i}}{4}$ $(i=1,2)$ | (4,2) | (4,2) |

Our qualitative result that it is not the minimal or the maximal hospital capacity which is optimal holds for a wide range of economic situations. We can imagine economies with one sector being dominant, for example, industrial production ($10g_{3}$), and the hospital costs are lower (supposing $\frac{h_{i}}{4}$ $(i=1,2)$). Then the maximin was still attained at the strategy pair (4,2). Hence the maximin solution for Player 2 (the state) is 2 ($Y_{3}=80 000$). Increasing hospital costs fourfold or decreasing the weight of one sector to a tenth of its value would still not change the outcome of the game.

From the above examples, and further simulations we can conclude that, in general, the maximum hospital capacity is not the optimal solution for the game’s conflict. These different maximin solutions call attention to the following: Even if two countries have the same political objective, namely to provide hospital care to all needing patients, they may have different hospital extension strategies, according to their different economic structures.

# 2. Estimation of the chance of a pandemic outbreak by taking into account time constraints and recovery rates

Here we present the formalization and solution to the time-dependent branching process that describes the growth of infectious individuals. The equations here allow us to compute the probability of spreading the disease to other countries.

Now we assume that the reproduction process $\xi\left( t \right)$is a non-homogenous Poisson point process with intensity function $\lambda\left( t \right)$. The random lifetime is denoted by $\eta$; for the sake of simplicity, we assume that the reproduction process is independent of $\eta$, apart that no reproduction ($=$ infection) is allowed beyond the lifetime. The survival function of $\eta$ will be denoted by $L\left( t \right)\mathbb{:=P(}\eta>t)$. Let $\mu(t)$ denote the expected number of offspring up to time $t$, that is, $\mu\left( t \right)\mathbb{=E}\left( \xi[0,t\wedge\eta] \right)$ where $\wedge$ stands for minimum. Then

$$\mu\left( t \right)\mathbb{=E}\left( \mathbb{E}\left( \xi[0,t\wedge\eta] | \eta\right) \right)\mathbb{=E}\int_{0}^{t\wedge\eta} \lambda\left( s \right)\text{d}s=\mathbb{E}\int_{0}^{t} \lambda\left( s \right)\mathbb{I}\text{(}\eta>s\text{)d}s,$$

where $\mathbb{I}\text{(}\cdot\text{)}$is the indicator of the event in brackets. Interchanging integration and expectation we get

$$\mu\left( t \right)=\int_{0}^{t} \lambda\left( s \right)\mathbb{P}\text{(}\eta>s\text{)d}s=\int_{0}^{t} \lambda\left( s \right)L(s\text{)d}s.$$

The expected total number of infected individuals is

$$R_{0}=\mu\left( +\infty\right)=\int_{0}^{+\infty} \lambda\left( s \right)L(s\text{)d}s.$$

We suppose that this is finite and greater than $1$, that is, our branching process is supercritical. Then the probability $z_{0}$ that the process, starting with a single infectious individual, will eventually terminate is the smallest positive solution of the equation $g\left( z \right)=z$, where $g\left( z \right)$ is the probability generating function of $\xi[0,\eta)$, that is,

$$g\left( z \right)=\mathbb{E}\left( z^{\xi[0,\eta]} \right)\mathbb{=E}\left( \mathbb{E}\left( z^{\xi[0,\eta]} | \eta\right) \right)\mathbb{=E}\left( \exp\left\{ -\int_{0}^{\eta} \lambda\left( s \right)\text{d}s(1-z) \right\} \right)\mathbb{=E}\left( e^{-\Lambda(\eta)(1-z)} \right),$$

where $\Lambda\left( t \right)=\int_{0}^{t} \lambda\left( s \right)\text{d}s$. Since $f\left( t \right)=1-e^{-\Lambda(t)(1-z)}$ is increasing and $f\left( 0 \right)=0$, we can write

$$g\left( z \right)=1-\mathbb{E}f\left( \eta\right)=1-\int_{0}^{+\infty} f^{'}\left( s \right)L\left( s \right)\text{d}s=1-\left( 1-z \right)\int_{0}^{+\infty} e^{-\Lambda\left( s \right)\left( 1-z \right)}\lambda\left( s \right)L\left( s \right)\text{d}s.$$

Consequently, the extinction probability $z_{0}$ satisfies

$$1=\int_{0}^{+\infty} e^{-\Lambda\left( s \right)\left( 1-z \right)}\lambda\left( s \right)L\left( s \right)\text{d}s.$$

Sometimes it is more convenient to introduce and use the complementary probability: the probability $w=1-z_{0}$ of a prolonging epidemic. It is the only positive solution to the equation

$$1=\int_{0}^{+\infty} e^{-\Lambda\left( s \right)w}\lambda\left( s \right)L\left( s \right)\text{d}s.$$

If the epidemic process starts with a single ancestor of age $t$ (that is, it was infected $t$ time ago), then the probability of the epidemic to terminate is $g_{t}(z_{0})$, where

$$g_{t}\left( z \right)=\mathbb{E}\left( z^{\xi(t,\eta]} | \eta>t \right)=\frac{1}{L(t)}\mathbb{E}\left( e^{-\left( \Lambda\left( \eta\right)-\Lambda\left( t \right) \right)(1-z)}\mathbb{I}\text{(}\eta>t\text{)} \right).$$

The same line of reasoning as above gives

$$g_{t}\left( z \right)=\frac{1}{L(t)}\left( 1-(1-z)\int_{t}^{+\infty} e^{-\left( \Lambda\left( s \right)-\Lambda\left( t \right) \right)\left( 1-z \right)}\lambda\left( s \right)L\left( s \right)\text{d}s \right).$$

On the complementary event the number of infecting individuals grows exponentially fast. The exponent $\varrho$ is called the Malthusian parameter, and it is the positive solution of the equation

$$\int_{0}^{+\infty} e^{-\varrho t}\text{d}\mu(t)=1,$$

that is,

$$\int_{0}^{+\infty} e^{-\varrho t}\lambda\left( t \right)L(t\text{)}\text{d}t=1.$$

In the sequel we will confine ourselves to the event of non-extinction. Let $Z(u)$ denote the number of individuals present in the process at time $u$. Then

$$e^{-\varrho u}\mathbb{E}Z\left( u \right)\to\frac{\int_{0}^{+\infty} e^{-\varrho s}L\left( s \right)\text{d}s}{\int_{0}^{+\infty} {s e}^{-\varrho s}\text{d}\mu(s)}=\frac{\int_{0}^{+\infty} e^{-\varrho s}L\left( s \right)\text{d}s}{\int_{0}^{+\infty} {s e}^{-\varrho s}\lambda\left( s \right)L(s\text{)d}s} .$$

It is also known (e.g. Theorem 6.4 of Nerman [^56^](#_ENREF_56)) that the proportion of individuals older than $t$ at time $u$ converges to

$$\frac{\int_{t}^{+\infty} e^{-\varrho s}L\left( s \right)\text{d}s}{\int_{0}^{+\infty} e^{-\varrho s}L\left( s \right)\text{d}s}$$

as $u\to+\infty$ (asymptotic age distribution).

Suppose a mutant virus appears in a country with $N$ residents, launching an epidemic. Recognizing the epidemic and starting to control it by appropriate restrictive measures requires time $T_{C}$. During this period, $U_{C}$ of the residents travel abroad. Let us compute the probability that the epidemic would spread abroad.

We suppose that this $U_{C}$ travelers leave their country uniformly during the period considered. The probability that an infectious traveller leaving her/his homeland at (a sufficiently large) time $u$ inflicts an epidemic that eventually terminates is (approximately) equal to

$$\frac{\int_{0}^{+\infty} e^{-\varrho s}L\left( s \right)g_{s}\left( z_{0} \right) \text{d}s}{\int_{0}^{+\infty} e^{-\varrho s}L\left( s \right)\text{d}s} ,$$

the average of the extinction probabilities with respect to the age distribution. The probability that in a small time-interval $[u, u+\Delta u]$ an infectious traveler crosses the border is

$$\frac{U_{C}}{N}Z\left( u \right)\Delta u\approx\frac{U_{C}}{N}e^{\varrho u}\frac{\int_{0}^{+\infty} e^{-\varrho s}L\left( s \right)\text{d}s}{\int_{0}^{+\infty} {s e}^{-\varrho s}\lambda\left( s \right)L(s\text{)d}s} \Delta u,$$

and the probability that she/he unleashes an enduring epidemic is approximately

$$\pi_{u}:=\frac{U_{C}}{N}e^{\varrho u}\frac{\int_{0}^{+\infty} e^{-\varrho s}L\left( s \right)\text{d}s}{\int_{0}^{+\infty} {s e}^{-\varrho s}\lambda\left( s \right)L(s\text{)d}s}\left( 1-\frac{\int_{0}^{+\infty} e^{-\varrho s}L\left( s \right)g_{s}\left( z_{0} \right) \text{d}s}{\int_{0}^{+\infty} e^{-\varrho s}L\left( s \right)\text{d}s} \right) \Delta u=\frac{U_{C}}{N}e^{\varrho u}\frac{\int_{0}^{+\infty} e^{-\varrho s}L\left( s \right)\left( 1-g_{s}\left( z_{0} \right) \right) \text{d}s}{\int_{0}^{+\infty} {s e}^{-\varrho s}\lambda\left( s \right)L(s\text{)d}s} \Delta u.$$

Since ${U_{C}}/N$ is typically small, so is $\pi_{u}$. Therefore, the probability of spreading the disease is

| $1-\prod_{u} \left( 1-\pi_{u} \right)\approx1-\exp\left\{ -\sum_{u} \pi_{u} \right\}\approx1-\exp\left\{ -\frac{U_{C}}{N}\cdot\frac{\int_{0}^{+\infty} e^{-\varrho s}L\left( s \right)\left( 1-g_{s}\left( z_{0} \right) \right) \text{d}s}{\int_{0}^{+\infty} {s e}^{-\varrho s}\lambda\left( s \right)L(s\text{)d}s}\int_{0}^{T_{C}} e^{\varrho u}\text{d}u \right\}=1-\exp\left\{ -\frac{U_{C}}{N}\cdot\frac{\int_{0}^{+\infty} e^{-\varrho s}L\left( s \right)\left( 1-g_{s}\left( z_{0} \right) \right) \text{d}s}{\int_{0}^{+\infty} {s e}^{-\varrho s}\lambda\left( s \right)L(s\text{)d}s}\cdot\frac{1}{\varrho}\left( e^{\varrho T_{C}}-1 \right) \right\}.$ | (SI.2) |
| --- | --- |

If $\varrho T_{C}\gg\log\left( N/{U_{C}} \right)$, the new disease will spread abroad with a very high probability. In addition, since the right-hand side is a double exponential function of the delay time $T_{C}$, a threshold phenomenon can be observed: the probability of the epidemic to spread abroad jumps from very small positive values to numbers close to $1$ during a relatively short period.

In our model the distribution of $\eta$ is the following.

| Value of $\eta$ | $t_{0}+t_{1}$ | $t_{0}+t_{1}+t_{2}$ |
| --- | --- | --- |
| Probability | $\theta_{1}$ | $1-\theta_{1}$ |

Hence the survival function is

| $t\in$ | $[0,t_{0}+t_{1})$ | $[t_{0}+t_{1},t_{0}+t_{1}+t_{2})$ | [$t_{0}+t_{1}+t_{2}, +\infty)$ |
| --- | --- | --- | --- |
| $L\left( t \right)$ | $1$ | $1-\theta_{1}$ | $0$ |

The intensity function $\lambda\left( t \right)$ of the reproduction process is

| $t\in$ | $[0,t_{0})$ | $[t_{0},t_{0}+t_{1})$ | $[t_{0}+t_{1},t_{0}+t_{1}+t_{2})$ | [$t_{0}+t_{1}+t_{2}, +\infty)$ |
| --- | --- | --- | --- | --- |
| $\lambda\left( t \right)$ | $0$ | $r_{1}$ | $r_{2}$ | $0$ |

Condition of supercriticality: $r_{1}t_{1}+\left( 1-\theta_{1} \right)r_{2}t_{2}>1$.

Equation for the probability $w$ of prolonging epidemic:

$$1=w+\theta_{1}e^{{-r}_{1}t_{1}w}+\left( 1-\theta_{1} \right)e^{-\left( r_{1}t_{1}+r_{2}t_{2} \right)w}, w>0.$$

The probability that an individual having been infected for time $t$ inflicts a *terminating* epidemic is $h_{t}\left( w \right)=g_{t}(1-w)$, where

| $h_{t}(w)=\left\{ \left. \begin{matrix} 1-w \\ {(1-w)e}^{r_{1}\left( t-t_{0} \right)w} \\ e^{-r_{2}\left( t_{0}+t_{1}+t_{2}-t \right)w} \end{matrix} \right. \right.$ |  | if | $t\in[0,t_{0})$, |
| --- | --- | --- | --- |
|  |  | if | $t\in[t_{0},t_{0}+t_{1})$, |
|  |  | if | $t\in\left[ t_{0}+t_{1},t_{0}+t_{1}+t_{2} \right).$ |

The Malthusian equation is

$$1=\int_{t_{0}}^{t_{0}+t_{1}} e^{-\varrho t}r_{1}\text{d}t+\int_{t_{0}+t_{1}}^{t_{0}+t_{1}+t_{2}} e^{-\varrho t}\left( 1-\theta_{1} \right)r_{2}\text{d}t$$

that is, $\varrho=A_{0}+A_{1}+A_{2}$, where

| $A_{0}=r_{1}e^{-\varrho t_{0}},$ |
| --- |
| $A_{1}=\left( \left( 1-\theta_{1} \right)r_{2}-r_{1} \right)e^{-\varrho\left( t_{0}+t_{1} \right)},$ |
| $A_{2}=-\left( 1-\theta_{1} \right)r_{2}e^{-\varrho\left( t_{0}+t_{1}+t_{2} \right)}.$ |

Next, let us compute $\int_{0}^{+\infty} {s e}^{-\varrho s}\lambda\left( s \right)L(s\text{)d}s$ and $\int_{0}^{+\infty} e^{-\varrho s}L\left( s \right)\left( 1-g_{s}\left( w \right) \right) \text{d}s$, the denominator and the numerator, resp., of the fraction in the exponent in Equation (SI.2).

$$\int_{0}^{+\infty} {s e}^{-\varrho s}\lambda\left( s \right)L(s\text{)d}s=r_{1}\int_{t_{0}}^{t_{0}+t_{1}} {s e}^{-\varrho s}\text{d}s+\left( 1-\theta_{1} \right)r_{2}\int_{t_{0}+t_{1}}^{t_{0}+t_{1}+t_{2}} s e^{-\varrho s}\text{d}s=A_{0}\left( \frac{t_{0}}{\varrho}+\frac{1}{\varrho^{2}} \right)+A_{1}\left( \frac{t_{0}+t_{1}}{\varrho}+\frac{1}{\varrho^{2}} \right)+A_{2}\left( \frac{t_{0}+t_{1}+t_{2}}{\varrho}+\frac{1}{\varrho^{2}} \right)=\frac{1}{\varrho}\left( 1+A_{0}t_{0}+A_{1}\left( t_{0}+t_{1} \right)+A_{2}\left( t_{0}+t_{1}+t_{2} \right) \right)$$

and

$$\int_{0}^{+\infty} e^{-\varrho s}L\left( s \right)\left( 1-g_{s}\left( w \right) \right) \text{d}s=w\int_{0}^{t_{0}} e^{-\varrho s} \text{d}s+\int_{t_{0}}^{t_{0}+t_{1}} e^{-\varrho s}\left( 1-{(1-w)e}^{r_{1}\left( s-t_{0} \right)w} \right)\text{d}s+\left( 1-\theta_{1} \right)\int_{t_{0}+t_{1}}^{t_{0}+t_{1}+t_{2}} e^{-\varrho s}e^{-r_{2}\left( t_{0}+t_{1}+t_{2}-s \right)w}\text{d}s$$

$$=w\frac{1-e^{-\varrho t_{0}}}{\varrho}+e^{-\varrho t_{0}}\frac{1-e^{-\varrho t_{1}}}{\varrho}-(1-w)e^{-\varrho t_{0}}\frac{e^{\left( r_{1}w-\varrho\right)t_{1}}-1}{r_{1}w-\varrho}+\left( 1-\theta_{1} \right)e^{-\varrho\left( t_{0}+t_{1}+t_{2} \right)}\frac{e^{\left( \varrho-r_{2}w \right)t_{2}}-1}{\varrho-r_{2}w} .$$

# 3. Derivation of the venue dependent transmission probability

The transmission probability expressed in Equation (1) of the main text is derived in the following. Human-to-human transmission via respiratory droplets (and to some extent aerosols) depends on the density of people at certain places, the time they spend at those places, and the intrinsic transmissibility rate of the pathogen. In essence, there are diverse types of habitats for the pathogen in which its transmission is different. We consider different types of venues, broad categories of places people frequent, i.e. shops and stores, entertainment, tourist attractions, offices, factories, etc. By introducing this type of heterogeneity into our model, we are able to different types of societies as well as non-pharmaceutical interventions mandated by the state. The limiting of access to certain venues (see below) is how a state can curb the spread of the pathogen.

Let us introduce the following notations.

- $n_{i}$ denotes the number of individuals on venue $i$, $i\in\left\{ 1,2,\ldots,H \right\}$.
- $k_{i}$ denotes the *interaction intensity:* A focal individual in venue $i$ is supposed to interact with $k_{i}$ people during one time period (15 minutes in our case). In other words, $k_{i}$ is the maximal number of susceptible people to whom the disease can be transmitted by an infected individual in one time period (handshake, being in a distance of less than two meters, sneezing, etc.)
- $\alpha_{i}$ denotes the probability of a susceptible person getting infected if exposed to the infection, in venue $i$, during one time period (15 minutes in our case). This depends on the size of the indoor place, ventilation, air flow, etc. It does not depend on the number of infectious persons (provided it is not zero) among the $k_{i}$ people in the neighbourhood of the focal individual (e.g., if the disease is highly contagious, the exposure to infection is high even for a single infectious interaction).
- $\tau_{i}$ denotes the number of time periods a person spends on average in venue $i$ during one day (the length of one period is 15 minutes in our case). Sometimes it is called the repetition number.

Now, what is the probability that in the neighborhood of the focal individual there is at least one infectious person?

In a well-mixed population the distribution of the (random) number of infected persons among $n_{i}$ people is hypergeometric, but it can be approximated by the binomial distribution with parameters $n_{i}$ and $q_{i}=\frac{I_{\mathrm{ps}}+I_{s}}{S+E+I_{\mathrm{ps}}+I_{s}+R}$ , because the number of people in venue $i$ is less than the size of the whole population by several orders of magnitude. Suppose there are $f$ infected individuals present. Consider the complementary event, that is, no one of the $k_{i}$ interacting people is infected. This has (conditional) probability

$$\frac{\left( \begin{matrix} n_{i}-f \\ k_{i} \end{matrix} \right)}{\left( \begin{matrix} n_{i} \\ k_{i} \end{matrix} \right)} .$$

By the law of total probability, we get for the complementary probability $p_{i}$ that

$$p_{i}=\sum_{f=0}^{n_{i}} \left( \begin{matrix} n_{i} \\ f \end{matrix} \right)q_{i}^{f}\left( 1-q_{i} \right)^{n_{i}-f}\frac{\left( \begin{matrix} n_{i}-f \\ k_{i} \end{matrix} \right)}{\left( \begin{matrix} n_{i} \\ k_{i} \end{matrix} \right)}=\left( 1-q_{i} \right)^{k_{i}}\sum_{f=0}^{n_{i}} \left( \begin{matrix} n_{i}-k_{i} \\ f \end{matrix} \right)q_{i}^{f}\left( 1-q_{i} \right)^{n_{i}-k_{i}-f}$$

$$=\left( 1-q_{i} \right)^{k_{i}}=\left( \frac{S+E+R}{S+E+I_{\mathrm{ps}}+I_{s}+R} \right)^{k_{i}}.$$

If the proportion of infectious people in the whole population is negligible compared to the number of susceptible or recovered (e.g. in the expansion period or when the herd immunity is close to be achieved), then an exponential function approximation can be used:

$$p_{i}\approx\exp\left( -\frac{\left( I_{\mathrm{ps}}+I_{s} \right)k_{i}}{S+E+I_{\mathrm{ps}}+I_{s}+R} \right).$$

A susceptible person gets infected during a unit period of time with probability $\alpha_{i}\left( 1-p_{i} \right)$, and gets off with probability $1-\alpha_{i}\left( 1-p_{i} \right)$. As the former quantity is usually not too small, here we cannot always switch to the exponential approximation.

Finally, how can a susceptible person not get infected? Only if neither of the $\tau_{i}$ occasions result in infection. Thus, the probability of acquiring the disease in venue $i$ is

$$\beta_{i}=1-\left( 1-\alpha_{i}\left( 1-p_{i} \right) \right)^{\tau_{i}}.$$

The probability of a susceptible person getting infected during one unit of time, that is, during one day equals

$$\beta=1-\prod_{i=1}^{H} \left( 1-\beta_{i} \right)=1-\prod_{i=1}^{H} \left( 1-\alpha_{i}\left( 1-p_{i} \right) \right)^{\tau_{i}}.$$

leading to Equation (1). If the quantities $\alpha_{i}\left( 1-p_{i} \right)$ are small enough, we can use exponential approximation again:

$$\beta\approx1-\exp\left\{ -\sum_{i=1}^{H} \tau_{i}\alpha_{i}\left( 1-p_{i} \right) \right\} .$$

# References

1 Fraser, C., Riley, S., Anderson, R. M. & Ferguson, N. M. Factors that make an infectious disease outbreak controllable. *PNAS* **101**, 6146–6151, doi:10.1073/pnas.0307506101 (2004).

2 Hayward, A. C. *et al.* Comparative community burden and severity of seasonal and pandemic influenza: results of the Flu Watch cohort study. *The Lancet Respiratory Medicine* **2**, 445–454, doi:10.1016/S2213-2600(14)70034-7 (2014).

3 Furuya-Kanamori, L. *et al.* Heterogeneous and dynamic prevalence of asymptomatic influenza virus infections. *Emerging Infect. Dis.* **22**, 1052–1056, doi:10.3201/eid2206.151080 (2016).

4 Ferguson, N. M., Mallett, S., Jackson, H., Roberts, N. & Ward, P. A population-dynamic model for evaluating the potential spread of drug-resistant influenza virus infections during community-based use of antivirals. *J. Antimicrob. Chemother.* **51**, 977–990, doi:10.1093/jac/dkg136 (2003).

5 CDC. *Disease Burden of Influenza*, 2020. https://www.cdc.gov/flu/about/burden/index.html

6 Liu, J. *et al.* Community transmission of Severe Acute Respiratory Syndrome Coronavirus 2, Shenzhen, China, 2020. *Emerging Infectious Disease journal* **26**, 1320–1323, doi:10.3201/eid2606.200239 (2020).

7 Backer, J. A., Klinkenberg, D. & Wallinga, J. Incubation period of 2019 novel coronavirus (2019-nCoV) infections among travellers from Wuhan, China, 20–28 January 2020. *Eurosurveillance* **25**, 2000062, doi:10.2807/1560-7917.ES.2020.25.5.2000062 (2020).

8 Li, Q. *et al.* Early transmission dynamics in Wuhan, China, of novel coronavirus–infected pneumonia. *New Engl. J. Med.* **382**, 1199–1207, doi:10.1056/NEJMoa2001316 (2020).

9 Linton, N. M. *et al.* Incubation period and other epidemiological characteristics of 2019 novel coronavirus infections with right truncation: A statistical analysis of publicly available case data. *Journal of Clinical Medicine* **9**, 538 (2020).

10 Guan, W.-j. *et al.* Clinical characteristics of coronavirus disease 2019 in China. *New Engl. J. Med.* **382**, 1708–1720, doi:10.1056/NEJMoa2002032 (2020).

11 Lauer, S. A. *et al.* The incubation period of coronavirus disease 2019 (COVID-19) from publicly reported confirmed cases: Estimation and application. *Annals of Internal Medicine* **172**, 577–582, doi:10.7326/m20-0504 (2020).

12 Bae, J.-M. A Chinese case of COVID-19 did not show infectivity during the incubation period: Based on an epidemiological survey. *Korean J Prev Med* **53**, 67–69, doi:10.3961/jpmph.20.048 (2020).

13 Pan, X. *et al.* Asymptomatic cases in a family cluster with SARS-CoV-2 infection. *The Lancet Infectious Diseases* **20**, 410–411, doi:10.1016/S1473-3099(20)30114-6 (2020).

14 Bai, Y. *et al.* Presumed asymptomatic carrier transmission of COVID-19. *JAMA* **323**, 1406–1407, doi:10.1001/jama.2020.2565 (2020).

15 Han, Y. & Yang, H. The transmission and diagnosis of 2019 novel coronavirus infection disease (COVID-19): A Chinese perspective. *J. Med. Virol.* **92**, 639–644, doi:10.1002/jmv.25749 (2020).

16 Huang, R., Xia, J., Chen, Y., Shan, C. & Wu, C. A family cluster of SARS-CoV-2 infection involving 11 patients in Nanjing, China. *The Lancet Infectious Diseases* **20**, 534–535, doi:10.1016/S1473-3099(20)30147-X (2020).

17 Kimball, A. *et al.* Asymptomatic and presymptomatic SARS-CoV-2 infections in residents of a long-term care skilled nursing facility — King County, Washington, March 2020. *MMWR Morb Mortal Wkly Rep* **69**, 377–381, doi:10.15585/mmwr.mm6913e1 (2020).

18 Zou, L. *et al.* SARS-CoV-2 viral load in upper respiratory specimens of infected patients. *New Engl. J. Med.* **382**, 1177-1179, doi:10.1056/NEJMc2001737 (2020).

19 Hu, Z. *et al.* Clinical characteristics of 24 asymptomatic infections with COVID-19 screened among close contacts in Nanjing, China. *Science China Life Sciences* **65**, 706–711, doi:10.1007/s11427-020-1661-4 (2020).

20 Wang, Y. *et al.* Clinical outcomes in 55 patients with severe acute respiratory syndrome coronavirus 2 who were asymptomatic at hospital admission in Shenzhen, China. *The Journal of Infectious Diseases* **221**, 1770–1774, doi:10.1093/infdis/jiaa119 (2020).

21 Ling, Z. *et al.* Asymptomatic SARS-CoV-2 infected patients with persistent negative CT findings. *European Journal of Radiology* **126**, doi:10.1016/j.ejrad.2020.108956 (2020).

22 Tian, S. *et al.* Characteristics of COVID-19 infection in Beijing. *The Journal of Infection* **80**, 401–406, doi:10.1016/j.jinf.2020.02.018 (2020).

23 Day, M. Covid-19: identifying and isolating asymptomatic people helped eliminate virus in Italian village. *BMJ* **368**, m1165, doi:10.1136/bmj.m1165 (2020).

24 Mizumoto, K., Kagaya, K., Zarebski, A. & Chowell, G. Estimating the asymptomatic proportion of coronavirus disease 2019 (COVID-19) cases on board the Diamond Princess cruise ship, Yokohama, Japan, 2020. *Eurosurveillance* **25**, 2000180, doi:10.2807/1560-7917.ES.2020.25.10.2000180 (2020).

25 Nishiura, H. *et al.* Estimation of the asymptomatic ratio of novel coronavirus infections (COVID-19). *International Journal of Infectious Diseases* **94**, 154–155, doi:10.1016/j.ijid.2020.03.020 (2020).

26 Park, S. Y. *et al.* Coronavirus disease outbreak in call center, South Korea. *Emerging Infect. Dis.* **26**, 1666–1670, doi:10.3201/eid2608.201274 (2020).

27 Byambasuren, O. *et al.* Estimating the extent of asymptomatic COVID-19 and its potential for community transmission: Systematic review and meta-analysis. *Official Journal of the Association of Medical Microbiology and Infectious Disease Canada* **COVID-19**, e20200030, doi:10.3138/jammi-2020-0030 (2020).

28 Zhou, X., Li, Y., Li, T. & Zhang, W. Follow-up of asymptomatic patients with SARS-CoV-2 infection. *Clin. Microbiol. Infect.* **26**, 957–959, doi:10.1016/j.cmi.2020.03.024 (2020).

29 Yu, P., Zhu, J., Zhang, Z. & Han, Y. A familial cluster of infection associated with the 2019 novel coronavirus indicating possible person-to-person transmission during the incubation period. *The Journal of Infectious Diseases*, doi:10.1093/infdis/jiaa077 (2020).

30 Ye, F. *et al.* Delivery of infection from asymptomatic carriers of COVID-19 in a familial cluster. *International Journal of Infectious Diseases*, doi:10.1016/j.ijid.2020.03.042 (2020).

31 Zhang, J., Tian, S., Lou, J. & Chen, Y. Familial cluster of COVID-19 infection from an asymptomatic. *Critical Care* **24**, 119, doi:10.1186/s13054-020-2817-7 (2020).

32 Ruan, Q., Yang, K., Wang, W., Jiang, L. & Song, J. Clinical predictors of mortality due to COVID-19 based on an analysis of data of 150 patients from Wuhan, China. *Intensive Care Medicine*, doi:10.1007/s00134-020-05991-x (2020).

33 Zhou, F. *et al.* Clinical course and risk factors for mortality of adult inpatients with COVID-19 in Wuhan, China: a retrospective cohort study. *The Lancet* **395**, 1054–1062, doi:10.1016/S0140-6736(20)30566-3 (2020).

34 Wang, D. *et al.* Clinical characteristics of 138 hospitalized patients with 2019 Novel Coronavirus–infected pneumonia in Wuhan, China. *JAMA* **323**, 1061–1069, doi:10.1001/jama.2020.1585 (2020).

35 Report of the WHO-China Joint Mission on Coronavirus Disease 2019 (COVID-19). (WHO, 2020).

36 ECDC. Coronavirus disease 2019 (COVID-19) in the EU/EEA and the UK – eighth update. (ECDC, 2020).

37 Riou, J. & Althaus, C. L. Pattern of early human-to-human transmission of Wuhan 2019 novel coronavirus (2019-nCoV), December 2019 to January 2020. *Eurosurveillance* **25**, 2000058, doi:10.2807/1560-7917.ES.2020.25.4.2000058 (2020).

38 Zhao, S. *et al.* Estimating the unreported number of novel coronavirus (2019-nCoV) cases in China in the first half of January 2020: A data-driven modelling analysis of the early outbreak. *Journal of Clinical Medicine* **9**, 388 (2020).

39 Majumder, M. & Mandl, K. D. Early Transmissibility Assessment of a Novel Coronavirus in Wuhan, China. *SSRN*, doi:10.2139/ssrn.3524675 (2020).

40 Zhao, S. *et al.* Preliminary estimation of the basic reproduction number of novel coronavirus (2019-nCoV) in China, from 2019 to 2020: A data-driven analysis in the early phase of the outbreak. *International Journal of Infectious Diseases* **92**, 214–217, doi:10.1016/j.ijid.2020.01.050 (2020).

41 Zhang, S. *et al.* Estimation of the reproductive number of novel coronavirus (COVID-19) and the probable outbreak size on the Diamond Princess cruise ship: A data-driven analysis. *International Journal of Infectious Diseases* **93**, 201–204, doi:10.1016/j.ijid.2020.02.033 (2020).

42 Zhou, T. *et al.* Preliminary prediction of the basic reproduction number of the Wuhan novel coronavirus 2019-nCoV. *Journal of Evidence-Based Medicine* **13**, 3–7, doi:10.1111/jebm.12376 (2020).

43 Long, Q. X. *et al.* Clinical and immunological assessment of asymptomatic SARS-CoV-2 infections. *Nat. Med.* **26**, 1200–1204, doi:10.1038/s41591-020-0965-6 (2020).

44 Wajnberg, A. *et al.* Robust neutralizing antibodies to SARS-CoV-2 infection persist for months. *Science* **370**, 1227–1230, doi:10.1126/science.abd7728 (2020).

45 May, R. M. *et al.* Epidemiology, transmission dynamics and control of SARS: the 2002–2003 epidemic. *Philosophical Transactions of the Royal Society of London. Series B: Biological Sciences* **359**, 1091–1105, doi:10.1098/rstb.2004.1490 (2004).

46 Cheng, V. C. C., Chan, J. F. W., To, K. K. W. & Yuen, K. Y. Clinical management and infection control of SARS: lessons learned. *Antiviral Res.* **100**, 407–419, doi:10.1016/j.antiviral.2013.08.016 (2013).

47 Hui, D. S. C. & Zumla, A. Severe Acute Respiratory Syndrome: Historical, Epidemiologic, and Clinical Features. *Infect Dis Clin North Am* **33**, 869–889, doi:10.1016/j.idc.2019.07.001 (2019).

48 Donnelly, C. A. *et al.* Epidemiological determinants of spread of causal agent of severe acute respiratory syndrome in Hong Kong. *The Lancet* **361**, 1761–1766, doi:10.1016/S0140-6736(03)13410-1 (2003).

49 Leung, G. M. *et al.* The epidemiology of severe acute respiratory syndrome in the 2003 Hong Kong rpidemic: An analysis of all 1755 patients. *Annals of Internal Medicine* **141**, 662–673, doi:10.7326/0003-4819-141-9-200411020-00006 (2004).

50 Zeng, G., Xie, S.-Y., Li, Q. & Ou, J.-M. Infectivity of severe acute respiratory syndrome during its incubation period. *Biomed Environ Sci* **22**, 502–510, doi:10.1016/S0895-3988(10)60008-6 (2009).

51 Riley, S. *et al.* Transmission dynamics of the etiological agent of SARS in Hong Kong: Impact of public health interventions. *Science* **300**, 1961–1966, doi:10.1126/science.1086478 (2003).

52 Chen, C.-Y. *et al.* Clinical features and outcomes of severe acute respiratory syndrome and predictive factors for acute respiratory distress syndrome. *Journal of the Chinese Medical Association* **68**, 4–10, doi:10.1016/S1726-4901(09)70124-8 (2005).

53 Peiris, J. S. M. *et al.* Clinical progression and viral load in a community outbreak of coronavirus-associated SARS pneumonia: a prospective study. *Lancet (London, England)* **361**, 1767–1772, doi:10.1016/s0140-6736(03)13412-5 (2003).

54 Vu, H. T. *et al.* Clinical description of a completed outbreak of SARS in Vietnam, February-May 2003. *Emerging Infect. Dis.* **10**, 334–338, doi:10.3201/eid1002.030761 (2004).

55 Wald, A. *Statistical Decision Functions*. (John Wiley, 1950).

56 Nerman, O. On the convergence of supercritical general (C-M-J) branching processes. *Zeitschrift für Wahrscheinlichkeitstheorie und Verwandte Gebiete* **57**, 365–395, doi:10.1007/BF00534830 (1981).
